# Genetic Architecture of Heterophylly: Single and Multi-Leaf Genome-Wide Association Mapping in *Populus euphratica*

**DOI:** 10.3389/fpls.2022.870876

**Published:** 2022-06-15

**Authors:** Xuli Zhu, Fengshuo Sun, Mengmeng Sang, Meixia Ye, Wenhao Bo, Ang Dong, Rongling Wu

**Affiliations:** ^1^Center for Computational Biology, College of Biological Sciences and Technology, Beijing Forestry University, Beijing, China; ^2^National Engineering Laboratory for Tree Breeding, Beijing Forestry University, Beijing, China; ^3^Key Laboratory of Genetics and Breeding in Forest Trees and Ornamental Plants, Ministry of Education, Beijing Forestry University, Beijing, China; ^4^The Tree and Ornamental Plant Breeding and Biotechnology Laboratory of National Forestry and Grassland Administration, Beijing Forestry University, Beijing, China; ^5^Institute of Reproductive Medicine, Medical School, Nantong University, Nantong, China; ^6^Center for Statistical Genetics, Pennsylvania State University, Hershey, PA, United States

**Keywords:** heterophylly, leaf shape, geometric morphometrics, genome-wide association study (GWAS), *Populus euphratica*

## Abstract

Heterophylly is an adaptive strategy used by some plants in response to environmental changes. Due to the lack of representative plants with typical heteromorphic leaves, little is known about the genetic architecture of heterophylly in plants and the genes underlying its control. Here, we investigated the genetic characteristics underlying changes in leaf shape based on the model species, *Populus euphratica*, which exhibits typical heterophylly. A set of 401,571 single-nucleotide polymorphisms (SNPs) derived from whole-genome sequencing of 860 genotypes were associated with nine leaf traits, which were related to descriptive and shape data using single- and multi-leaf genome-wide association studies (GWAS). Multi-leaf GWAS allows for a more comprehensive understanding of the genetic architecture of heterophylly by considering multiple leaves simultaneously. The single-leaf GWAS detected 140 significant SNPs, whereas the multi-leaf GWAS detected 200 SNP-trait associations. Markers were found across 19 chromosomes, and 21 unique genes were implicated in traits and serve as potential targets for selection. Our results provide novel insights into the genomic architecture of heterophylly, and provide candidate genes for breeding or engineering *P. euphratica*. Our observations also improve understanding of the intrinsic mechanisms of plant growth, evolution, and adaptation in response to climate change.

## Introduction

Heterophylly refers to the changes of leaf shape and size along the axis within a single plant, and is regarded as phenotypic plasticity occurring in response to environmental cues, such as temperature, light intensity, hypoxia, and water availability (Pigliucci and Marlow, 2001). Heterophylly occurs in various plants, including terrestrial and aquatic species (Jaya et al., 2010; Leigh et al., 2011). Morphological characters include leaf shape, leaf size, the relative positions of leaves on the stem, the rate of leaf initiation, etc. these reflect important biological phenomena, including heteroblasty, anisophylly, phase transitions, and heterophylly (Poethig, 1988; Dengler, 1999; Costa et al., 2012). The leaf form of heterophylly shows different morphological features according to genetic variation and environmental factors (Ernande and Dieckmann, 2004). For example, short and long leaves indicate the vital functional significance of heterophylly in *Ginkgo biloba* (Leigh et al., 2011). In contrast to heteroblasty and anisophylly, heterophylly is represented as the cue-induced switch between two or more leaf morphologies. In some situations, heterophylly is thought to be an adaptive mechanism, and is therefore of ecological and evolutionary significance (Zotz et al., 2011).

Previous studies mainly focused on the morphological, anatomical, and physiological aspects of heterophylly (Kaplan, 1980; Lee and Richards, 1991). For example, physiological indexes, including gas exchange and water use, showed significant differences between different leaf types on a single plant (Kitajima et al., 1997). Many studies have suggested that various hormones are involved in the regulation of heterophylly (Kuwabara et al., 2003; Iida et al., 2009). For example, abscisic acid (ABA) was shown to regulate leaf shape, and ethylene affected the cell elongation and shape of leaves in *Ludwigia arcuata* (Kuwabara et al., 2003; Sato et al., 2008). Recently, Nakayama et al. (2017) reviewed how phytohormones regulate heterophylly, and attempted to assess the biochemical mechanisms underlying heterophylly. The basic mechanisms of heterophylly at the molecular level have been elucidated in model plants, such as *Arabidopsis thaliana* (Yang et al., 2013; Rubio-Somoza et al., 2014; Xu et al., 2019). Over the past decade, a key regulatory module consisting of miR156 and its targets, *the SQUAMOSA PROMOTER BINDING PROTEIN-LIKE* (*SPL*) genes, was shown to play important roles in diverse aspects of plant development, including phase transition, heterophylly, and heteroblasty (Wu et al., 2009; Poethig, 2013; Manuela and Xu, 2020). Several studies have revealed the molecular mechanisms underlying heterophylly in non-model plants (Nakayama et al., 2014; Sicard et al., 2014; Kim et al., 2018). Studies in *Rorippa aquatica* suggested that the *KNOTTED1-LIKE HOMEOBOX Class I* (*KNOX1*) genes changed leaf primordia through the accumulation of gibberellin (GA) in response to environmental stimuli (Nakayama et al., 2014). Fu et al. (2012) reported that the miR156-SPL module is associated with the development of heterophylly in woody plants. However, few studies have investigated the genetic architecture of heterophyllous variation at the level of the whole genome.

The genome-wide association study (GWAS) has evolved as a powerful tool to understand the genetic control of complex traits, and has been successfully used to detect a substantial number of candidate genes for leaf traits in plants (Tian et al., 2011; Zhao et al., 2018; Mähler et al., 2020; Ye et al., 2020; Ahmed et al., 2021; Fu et al., 2021). However, these GWAS studies used only descriptive traits of leaf shape, such as width and length, and neglected information about the spatial distribution of shape changes. Therefore, the geometric morphometrics (GM) model has been used to detect leaf shape changes, and precisely describes leaf variation (Adams et al., 2013). To better understand the genetic mechanisms underlying leaf architecture, two GM approaches, namely landmark-based and outline analyses, have been used for genetic mapping. This new statistical framework, called shape mapping, can map specific shape quantitative trait loci (QTLs) that affect leaf shape variation (Langlade et al., 2005; Fu et al., 2010; Bo et al., 2014). However, the above models for shape mapping cannot be used to characterize heterophylly variation, and these methods neglect the differentiation of leaf shape at multiple positions within the same plant. To resolve this problem, the principle of GM-based shape mapping was expanded to map QTLs for the shapes of leaves in various positions, and this method can test specific QTLs potentially mediating heterophyllous variation throughout the genome (Sun et al., 2018). These shape mapping tools are valuable for exploring the genetic architecture of heterophylly.

**FIGURE 1:**
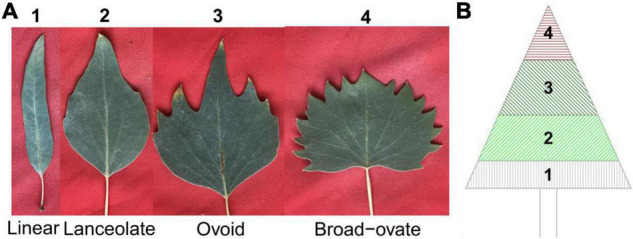
Types and spatial distribution pattern of heterophylly in *Populus euphratica*. **(A)** An example of the four types of heterophylly in *P. euphratica*. **(B)** The spatial distribution of four types of heterophylly in the canopy of mature in *P. euphratica*.

*Populus euphratica* Oliv. is a vital woody plant of riparian ecosystems in arid and semi-arid regions, and exhibits extremely high tolerance to abiotic stresses, especially salt stress (Chen et al., 2004; Ling et al., 2015). Leaf morphology is linear, lanceolate, ovate, and broad–ovate from the bottom to the top of the canopy in adult *P. euphratica* (Figure 1). This transition of leaf shape shows that it has typical heterophylly (Zhao and Qin, 2017; Zhai et al., 2020). To date, most research regarding heterophylly of *P. euphratica* has focused mainly on leaf morphology, anatomical structure, leaf development, and physiological level (Zhai et al., 2020; Song et al., 2021). Some recent studies explored the molecular mechanisms underlying the genesis of *P. euphratica* heteromorphic leaves through high-throughput sequencing analyses (Iqbal et al., 2017; Qin et al., 2018; Zeng et al., 2019; Guo et al., 2020; Li et al., 2020; Fu et al., 2021). These studies suggested that specific genes, miRNAs, lncRNAs, and circRNAs may interact with each other in the regulation of leaf morphogenesis in *P. euphratica* (Song et al., 2021). To date, however, the genetic mechanisms underlying heterophylly of *P. euphratica* have rarely been studied.

In this study, heteromorphic leaves were collected from 860 diverse *P. euphratica* genotypes, photographed, and phenotyped through image analysis. Whole-genome sequencing produced a dense marker set of more than 400,000 single-nucleotide polymorphisms (SNPs). We further evaluated population stratification based on SNPs covering the *P. euphratica* genome. We performed single-leaf association tests between descriptive measures and leaf outline heterophylly using a mixed linear model of correlated traits. We also performed a multi-leaf GWAS, to increase the power and identify SNPs and candidate genes involved in controlling the formation of heterophylly in *P. euphratica*. This comprehensive GWAS provided a better understanding of the genetic architecture of heterophylly, and will facilitate molecular marker-assisted breeding in *P. euphratica*. It also provided new insights into how heterophylly has responded to the harsh environment during the evolution of *P. euphratica*.

## Materials and Methods

### Plant Materials

The association natural population was composed of 860 *P. euphratica* genotypes, which were originally distributed in the Tarim River Basin of Yuli County, Xinjiang Uygur Autonomous Region, China (41°0′–41°2′N, 86°0′–86°2′E) (Supplementary Figure 1). The approximate age of the sampled trees varied from 50 to 100 years. Trees showing good growth and no disease or insect pests were randomly selected from within an area of 400 km^2^, and the distance between the sampled trees was at least 50 m to decrease kinship.

### Leaf Collection, Photography, and Image Analysis

Four types of heterophylly were classified on the basis of the leaf index (LI, defined as leaf length [LL]/leaf width [LW]): linear (LI ≥ 4), lanceolate (4 > LI ≥ 2), ovoid (2 > LI ≥ 1), and broad–ovate (LI < 1) (Figure 1A). Heteromorphic leaves were picked from the bottom to the top position of the entire canopy for each mature *P. euphratica* in July 2017 (Figure 1B). A total of 3,644 leaves were collected for subsequent photographs.

Leaves were photographed against a red background with the same focal distance by a digital camera (DXM 5600; Nikon). Digital image analysis was performed as follows:

(1) Each leaf picture was read into R software (ver. 4.0; R Core Team, 2021) by package *imager* (Simon, 2021) and the RGB matrix was extracted to distinguish the leaf from the background.
(2) The RGB matrix of the leaf was converted into a binary matrix to capture the leaf outline.
(3) The effects of the scale, position, and orientation of each type of heteromorphic leaf were adjusted for according to the method described by Fu et al. (2013), and the effect of leaf size was adjusted for by an orthogonal Procrustes process (Viscosi and Cardini, 2011).
(4) A set of semi-landmarks describing the leaf boundary were generated at equal radial angles, and the Cartesian coordinates of each point on the leaf boundary were calculated.

The image analysis process was performed in R, Supplementary Figure 2 shows the pipeline used for obtaining the leaf boundary from a leaf image. Finally, the descriptive data, including LL, LW, LI, and leaf area (LA), and the coordinate matrix delineating the leaf outline as phenotypic data, were used in subsequent analyses.

### Phenotypic Analysis

Descriptive statistics, normality testing, coefficient of variation (CV), and Spearman’s rank correlation analysis of the descriptive data of leaf shape were performed. The statistical significance of differences among the descriptive values of four types of leaf was evaluated by one-way analysis of variance (ANOVA). Elliptic Fourier (EF) analysis was used to model the closed outline with two-dimensional (*x*, *y*) coordinate data for each leaf (Sun et al., 2018). Multivariate analysis of variance (MANOVA) was used to examine the significance of differences among the outlines of four types of leaf with the estimated Fourier coefficients. Multiple comparisons were performed using the Bonferroni method. To improve the performance and computational efficiency of GM-based GWAS, principal component analysis (PCA) was used to reduce the dimensions. Each principal component (PC) can reflect the specific morphology of heterophylly. In this study, PCs explaining >96% of the cumulative variance were chosen. To evaluate the variance and residual explained by each PC based on the CV, the maximum absolute value was calculated for each PC, and a new PC was generated to ensure that all genotypes were represented in each PC. Box–Cox transformation to a normal distribution was performed for PCs that were not normally distributed. PCA was calculated using the *prcomp* function in R. Additional details about the PCA of leaf shape were described previously by Fu et al. (2013). All statistical analyses were performed using R 4.0.

### Genotypic Data

All *P. euphratica* genotypes were sequenced using the Illumina HiSeq 2000 platform (Illumina, San Diego, CA, United States). Clean data were obtained after removing low-quality bases and adapters from the raw data. Clean reads of the 860 genotypes were mapped to the *P. euphratica* reference genome (Ma et al., 2013) with BWA software (Li and Durbin, 2009) using the default parameters. The mapping results were generated in BAM format, and non-unique and unmapped reads were removed with SAMtools (Li et al., 2009). SNPs were called using GATK (McKenna et al., 2010) and were filtered by applying the following criteria in VCFtools (Danecek et al., 2011): mapping quality > 20; missing rate < 50%; minor allele frequency (MAF) < 5%; mean depth of coverage > 40; and *p*-value of Hardy–Weinberg equilibrium test > 0.05. Finally, 401,571 SNPs were screened for subsequent analysis. The genotype data has been deposited in the Genome Variation Map in National Genomics Data Center, under accession number GVM000321 that can be publicly accessible at https://bigd.big.ac.cn/gvm/getProjectFile?t=b9430bac.

### Population Structure

A Bayesian clustering approach implemented in the software *fastSTRUCTURE* (Raj et al., 2014) was used to investigate the population structure of *P. euphratica*. *K*-values (i.e., number of individual ancestry proportions) ranging from 2 to 15 were tested by 10 replicates per *K*, using the default convergence criterion and priors. A reasonable range of *K* values was determined by maximizing marginal likelihood and model components, and using the *chooseK.py* function in *fastSTRUCTURE*. We also performed PCA to further investigate the structural components of the *P. euphratica* population, and the relationships among them, using the R package *SNPRelate* (Zheng et al., 2012). The *King* program was used to calculate the kinship matrix (Manichaikul et al., 2010) among the 860 genotypes used in subsequent analysis. Linkage disequilibrium (LD) was evaluated using PLINK (Purcell et al., 2007) with the following parameters: –ld-window-r2 0 –ld-window 99999 –ld-window-kb 1000.

Decay curves were fitted using the non-linear least-squares method based on an exponential equation. LD decay plots were drawn using R 4.0.

### Single-Leaf Genome-Wide Association Studies

Let *Z*_*i*_ represent the descriptive phenotypic or PC value for a particular type of leaf from tree *i*. We used a linear mixed effect model to detect genomic regions controlling the shape of the leaf; this model is expressed as:


(1) Z=μ+X⁢α+P⁢β+η

where μ represents the intercept coefficient; *X* is a vector of SNP genotypes; α is a vector of the fixed effect of the SNP; *P* represents the population group; β is a vector of the fixed effect of the population structure; η is a random variable capturing the random polygenic effect, distributed as $\eta ∼\mathrm{MVN}\,\left(0,K\,\sigma_{g}^{2}\right)$, where *K* is the realized kinship matrix among different genotypes and $\sigma_{g}^{2}$ is the additive genetic variance; and ε is a vector of error, distributed as ε∼MVN(0,Iσ)e2.

In this study, different methods were used to test the associations between genotypes and leaf phenotypes. Equation (1) is called QK model. If the fixed effect of population structure and the random effect of kinship are neglected in equation (1), which is simplified as linear regression (LM) model. If only the random polygenic effect η is moved in equation (1), which is used as a Q model to analyze continuous quantitative traits. The Q–Q plot and genomic inflation factors were used to evaluate the optimal GWAS model. Permutation tests were used to determine the critical thresholds by reshuffling real phenotypes 1,000 times to break the connections between these phenotypes and their corresponding genotypes (Churchill and Doerge, 1994), and the 5% of 1,000 LR values is used as a critical threshold. The QK model was implemented in the R package *GMMAT* (Chen et al., 2020).

### Multi-Leaf Genome-Wide Association Studies

In contrast to traditional leaf GWAS models, the method used in this study involved mapping of high-dimensional phenotypes for heterophylly. The outline of each leaf was described by selecting a set of *L* semi-landmarks in the clockwise direction, at equally spaced radial angles on the leaf boundary. Let *Z*_*i*_ = (*z*_*i1*_, *z*_*i2*_, *z*_*i3*_, *z*_*i4*_) = ((*x*_*i1*_, *y*_*i1*_), (*x*_*i2*_, *y*_*i2*_), (*x*_*i3*_, *y*_*i3*_), (*x*_*i4*_, *y*_*i4*_)) = (((*x*_*i1*_(1), *y*_*i1*_(1)), … (*x*_*i1*_(*L*), *y*_*i1*_(*L*))), …, (((*x*_*i4*_(1), *y*_*i4*_(1)), … (*x*_*i4*_(*L*), *y*_*i4*_(*L*))))) represent the *x*- and *y*- coordinates of vectors of four types of heterophylly for an individual *i*. Let *C*_*i*_ = (*c*_*i1*_, *c*_*i2*_, *c*_*i3*_, *c*_*i4*_) denote the vectors of the phenotype and target PC of four heterophyllous leaves for an individual *i*. PCs are obtained by dimension reduction of the shape space coordinate *Z* with PCA. Assuming that there is a SNP that controls the formation of heterophylly, the likelihood of the phenotypes or PCs for a given SNP can be expressed as:


(2) $$L\,\left(C\right)=\prod_{j=1}^{J}\prod_{i}^{n_{j}}f_{j}\,\left(\left(c_{i\,1},c_{i\,2},c_{i\,3},c_{i\,4}\right)|{\text{Θ}}_{j},\eta \right)$$

where *J* is the number of SNP genotypes observed for the marker; *n*_*j*_ is the number of individuals of SNP genotype *j*; *f*_*j*_((*c*_*i*1_,*c*_*i*2_,*c*_*i*3_,*c*_*i*4_)|Θ_*j*_,η) is a multivariate normal density distribution of phenotypes for individual *i* at SNP genotype *j*, where Θ_*j*_ is a set of expectation parameters that model the mean vectors for SNP genotype *j*, and η is the variance-covariance parameter common to all genotype groups that generated the covariance matrix via the SAD (1) model (Zhao et al., 2005).

Through likelihood (2), the existence of heterophylly QTLs can be tested using the following hypotheses:


(3) H:0Θj,≡Θj=1,…,J

*H*_1_: At least one of the equalities aforementioned does not hold,

where *H*_0_ corresponds to the reduced model and *H*_1_ corresponds to the full model, in which there exists a heterophylly QTL. The above hypotheses were tested by comparing the log-likelihood ratios of the reduced and full models. An empirical approach based on permutation tests can be used to determine the thresholds for the hypotheses (Churchill and Doerge, 1994). The above model can also be extended by fully considering the influence of population structure.

### Functional Annotation

The SNP annotation and effect predictions were done using SnpEff software (Cingolani et al., 2012), based on the PopEup_1.0 genome sequences from the NCBI database (Ma et al., 2013). Genomic regions suitable for screening candidate genes were identified based on the gap between the left- and right-most significant SNPs in a Manhattan plot, and based on SNPs within ±100 kb of significant SNPs not inside the tower-like structure of the Manhattan plot. Candidate genes were annotated with the BLAST2GO tool (Conesa and Götz, 2008) using the non-redundant protein (Nr) database.

## Results

### Heterophylly Variation

**FIGURE 2:**
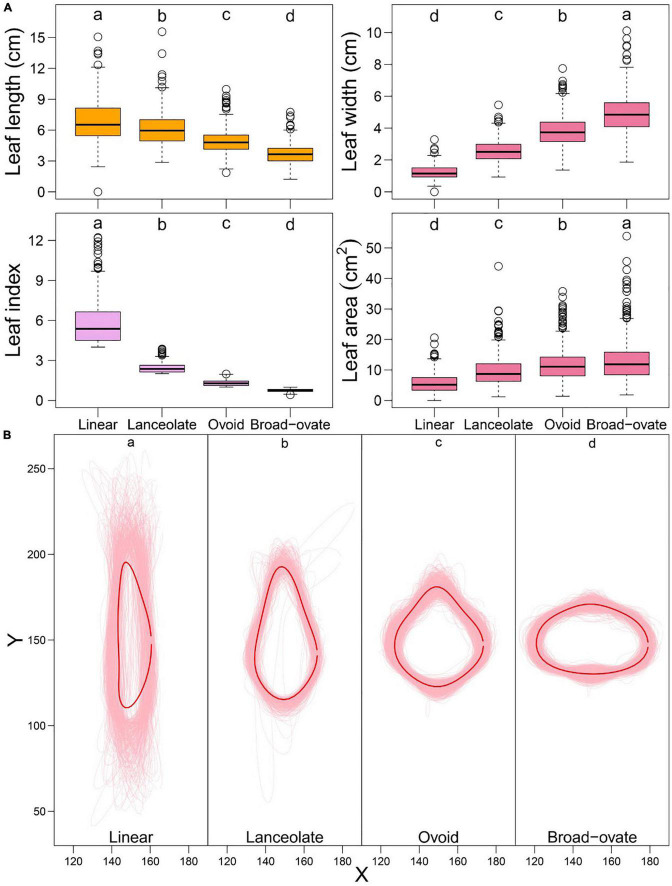
Descriptive data and shape distribution and MANOVA test for heterophylly, the letters (a, b, c, and d) indicate multiple comparison results at the significant level 0.05. **(A)** Descriptive data distribution and MANOVA for the descriptive data of heterophylly. **(B)** Shape distribution and MANOVA for the shape of heterophylly. The light pink thin line denotes the shape of heterophylly for each genotype, red thick line denotes the mean shape of all genotypes for four types of heterophylly.

ANOVA indicated that the means of each descriptive measure (LL, LW, LI, and LA) were statistically significantly different among the four types of heteromorphic leaf classes (Figure 2A). The leaf outlines of a population of 860 *P. euphratica* accessions were reconstructed based on different types of leaves through EF analysis (Figure 2B). MANOVA demonstrated significant differences in outlines among leaves (Figure 2B).

**FIGURE 3:**
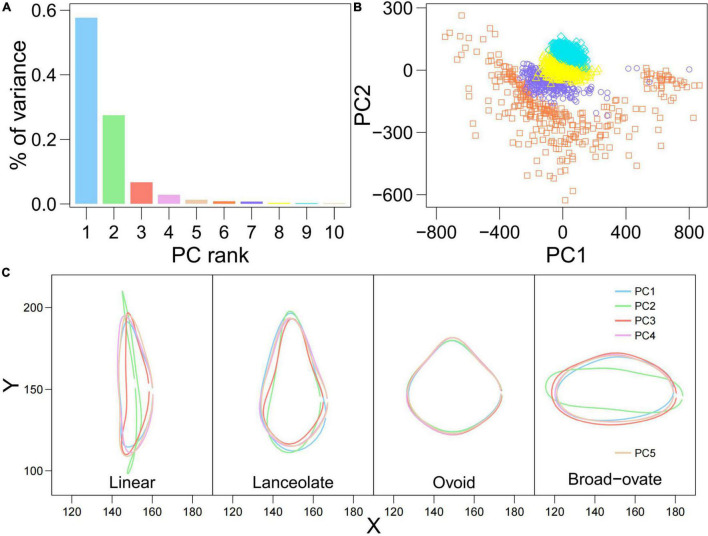
Principal component analysis (PCA) analysis calculated from the landmark data of 860 genotypes for heterophylly. **(A)** The barplot of PC contributions. **(B)** Biplot of the first and second principal components. The square, circle, triangle, and diamond symbols represent the linear, lanceolate, ovoid, and broad-ovate leaves, respectively. **(C)** The mean shape of four heterophyllous leaves on the first five PCs.

To examine the variation of heterophylly in the whole sample, PCA was applied to the superimposed coordinates of the leaf boundaries. PCA showed that the first five orthogonal axes together explained 96.1% of the variance in accessions (57.7, 27.5, 6.7, 2.9, and 1.3%, respectively) (Figure 3A). Clear overlap was seen between the linear and lanceolate leaves, and the ovoid and broad–ovate leaves, but the linear, lanceolate and ovoid, broad–ovate leaves were separated along the PC2 axis (Figure 3B). As shown in Figure 3C, the outlines of linear, lanceolate, and broad–ovate leaves exhibited significant variation among the five PCs. Although the morphologies of ovoid leaves were similar among the five PCs, there were significant differences in leaf tip and leaf base (Figure 3C).

To further evaluate the variation and distribution of phenotypes among individuals, descriptive statistical analysis was performed using R. The CVs of all traits showed an extensive distribution range in the population, but the CVs of all traits showed no specific patterns within a given leaf type (Supplementary Table 1). Interestingly, the largest CVs for LA were seen in heteromorphic leaves, ranging from 56 to 58%. In general, the CVs of phenotypes LL, LW, LI, and LA were significantly greater than those of the PCs (Supplementary Table 1). Skewness and kurtosis coefficients were obtained to assess the data distribution using the Shapiro–Wilk test. Kurtosis of the traits varied from 2.5 to 13.67, and skewness from −0.08 to 2.47. These two parameters deviated markedly from normality for most traits, as verified by the Shapiro–Wilk test (*P* < 0.01). The results of descriptive statistical analysis suggested that the extensive variation in leaf outline was due not only to the use of traditional leaf measurement methods, but also to the PCs.

We assessed the relationships among all traits (LL, LW, LI, and LA), and the PCs, by correlation analysis (Supplementary Figure 3). Generally, the four traits showed strong correlations with each other within the same leaf type (Supplementary Figure 3). However, strong negative associations were found in the five PCs for leaf outline. The phenotypes exhibited moderate relationships for heteromorphic leaves. Interestingly, the five PCs showed weak or no correlations across different types of leaves, suggesting that each PC may reflect a unique morphological trait of heteromorphic leaves. We also found a high degree of phenotypic similarity among some descriptive traits, and some PCs, within the same type of leaf. For example, the positive correlations of PC5 with LW, LI, and LA for linear shape, and the negative correlation of PC2 and LI (Supplementary Figure 3) for ovoid shape, suggest that some PCs may contain information about LL, LW, LI, and LA.

### Population Structure

Population structure was inferred by *fastSTRUCTURE* using all markers across the entire genome of 860 genotypes. The final number of subpopulations was two (*K* = 2), based on the marginal likelihood. There was a small degree of ancestral population stratification, although the majority of genotypes were classified into one “general” population (Supplementary Figure 4A). We performed a further PCA to investigate global genetic variation in the *P. euphratica* panel. The first two PCs accounted for 1.25 and 0.78% of the variation in the genotypic data, respectively (Supplementary Figure 4B). In particular, PC1 clearly distinguished the two subpopulations (Pop1 and Pop2). The *fastSTRUCTURE* and PCA results suggested that there was significant population structure difference among individuals of this natural population of *P. euphratica*. The LD decay with distance across the entire *P. euphratica* genome for all individuals was ∼61 Kb when the LD baseline was set to *r* = 0.1 (Supplementary Figure 4C). The LD decay observed in Pop1 was consistent with that of the whole population, but Pop2 showed a much slower rate of LD decay compared with both Pop1 and the entire population (Supplementary Figure 4C).

### Single-Leaf Genome-Wide Association Studies

As the data of most traits showed a skewed distribution (Supplementary Table 1), Box–Cox transformations were required to normalize the data and reduce false-positive signals in the GWAS. The Shapiro–Wilk test showed that all phenotypes were approximately normally distributed at *p* < 0.05 (Supplementary Figure 5). Three different models (LM, Q, and Q + K) were used to test the associations between single-leaf traits and the 401,571 SNP genotypes evaluated in our 860 *P. euphratica* accessions. The Q–Q plot from the GWAS indicated that the three methods showed similar performance for all traits (Supplementary Figure 6), suggesting that population structure and cryptic relatedness had little influence on the precision of the GWAS analysis of this natural population. Therefore, the LM model (excluding both the population structure and kinship matrix) was used to detect significant associations between leaf traits and SNPs.

**FIGURE 4:**
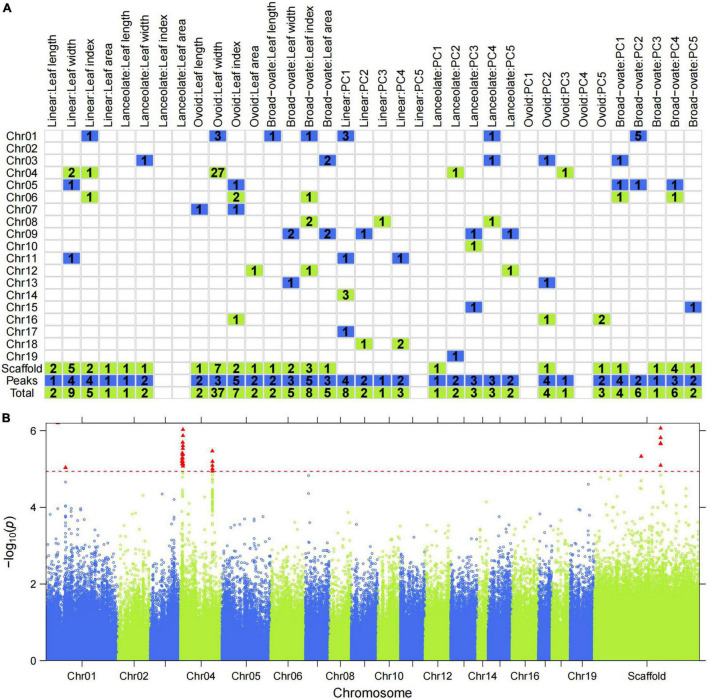
Genome-wide distribution of *P. euphratica* SNP loci detected based on single-leaf GWAS. **(A)** Distribution of the number of significant SNPs across each chromosome for all traits. **(B)** Manhattan plot displaying the GWAS result of the trait ‘Ovoid: Length width’ based on the LM model. The red triangle indicates the significantly associated SNPs. Redline indicates the threshold with permutation tests at 0.05.

We examined a total of 140 significant SNP-trait associations using stringent significance thresholds based on permutation (Supplementary Table 2). However, no significant associations were found for LI or LA in lanceolate leaves, or for PC5 in linear leaves, or PC1 or PC4 in ovoid leaves (Supplementary Table 2 and Figure 4A). The number of markers associated with each trait/PC ranged from 1 (LA and PC3 of linear leaves, LW and PC1 of lanceolate leaves, PC3 of ovoid leaves, and PC5 of broad–ovate leaves) to 37 (LW of ovoid leaves). These markers were distributed on 19 chromosomes and several unanchored scaffolds (Figure 4A). Furthermore, no significantly overlapping SNPs were observed between the different leaf types (Supplementary Figure 7A), whereas two significantly overlapping SNPs were detected between the descriptive traits (Supplementary Figure 7C). One SNP was associated with both the descriptive traits and PCs (Supplementary Figure 7B). We also examined significantly overlapping SNPs for the different PCs (Supplementary Figure 7D). Three SNPs that affected both PC1 and PC4 were tested. Of the significant SNPs, 64 (45.7%) were located in intergenic regions, while 46 (32.9%) were in upstream, downstream, or intronic regions; 14 (10%) of the significant SNPs were in non-coding areas of genes (Supplementary Table 2). In addition, six missense and seven synonymous point mutations were detected (Supplementary Table 2), which most likely affected protein function and contributed to leaf variation. We also examined how much of the phenotypic variation was explained by individual SNPs. Notably, 12 (8.6%) significant individual SNPs explained >8% of the observed phenotypic variation.

As an example, Figure 4B shows the association signals of all SNPs along the chromosomes for LW in ovoid leaves. Most significant SNPs converged to form a tower-like structure in the Manhattan plot (Figure 4B), indicating the presence of only a few genomic regions controlling the LW of ovoid leaves. Two powerful association signals were detected for this phenotype, forming two QTL clusters on chromosome 4. The first cluster (first tower-like region include 21 SNPs) was located in four scaffolds in NW_011501471.1, NW_011500351.1, NW_011500738.1, and NW_011500414.1, with a peak SNP (missense mutation) at 0.104 Mbp in NW_011500414.1 (*P* = 5.67e-07, Supplementary Table 2). A total of six significant SNPs in the second cluster were mapped to NW_011500574.1 and NW_011500101.1, and two synonymous SNPs (SNP296047 and SNP296048) were in the same genomic region, which may play an important role in regulating LW in ovoid leaves.

### Multi-Leaf Genome-Wide Association Studies

Single-leaf GWAS were performed to characterize phenotypic variation in individual leaves, but failed to capture the differentiation of leaf shape among multiple classes, limiting their application for determination of the more complex genetic mechanisms of heterophylly. Here, multi-leaf GWAS were used to identify the genomic loci potentially controlling heterophylly in *P. euphratica*. The results of single-leaf GWAS showed that the inclusion of population stratification and the kinship matrix did not substantially increase confounding for the descriptive traits and PCs, so their effects were ignored in multi-leaf GWAS.

**FIGURE 5:**
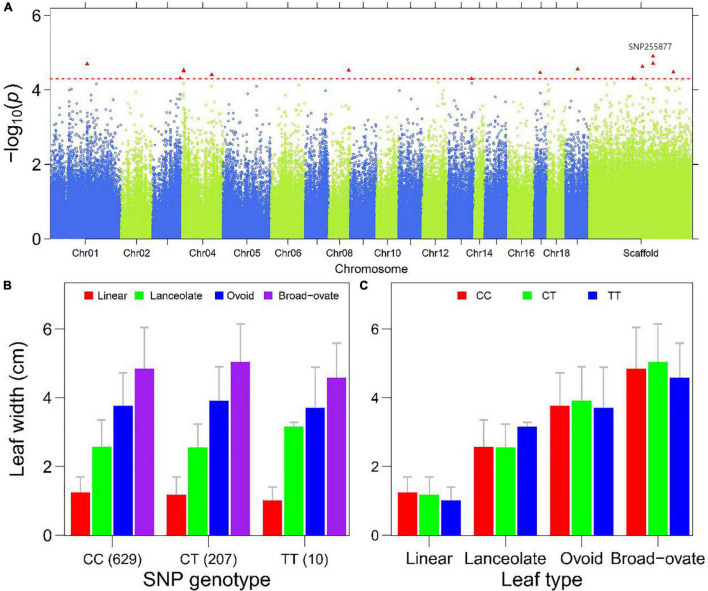
Genome-wide association mapping of the leaf width in *P. euphratica*. **(A)** Manhattan plot displaying the GWAS result of the trait ‘leaf width’ based on the single-leaf GWAS. The significantly associated SNP markers are labeled. **(B)** Histograms of leaf width of different leaf types plotted as a function of genotypes at SNP255877. **(C)** Histograms of allelic effects of SNP255877 for the heterophylly.

**FIGURE 6:**
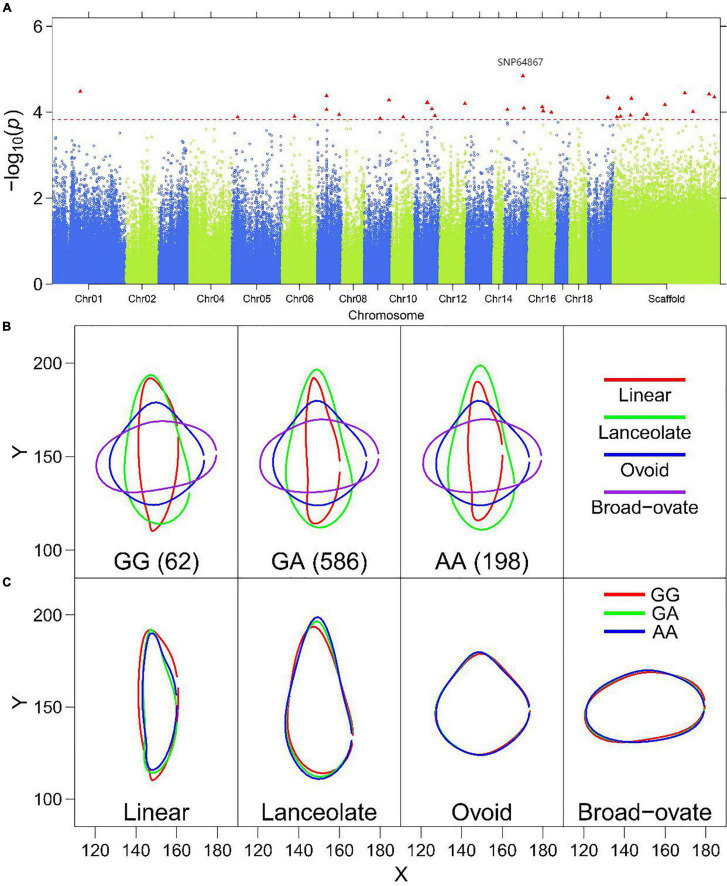
Multi-leaf GWAS for the PC1 of leaf structure in *P. euphratica*. **(A)** Manhattan plot displaying the GWAS result of the PC1 based on the multi-leaf GWAS. The significantly associated SNP markers are labeled. **(B)** Leaf outline of PC1 of heterophylly plotted as a function of genotypes at SNP64867. **(C)** Visualized variation in the shape of four heterophyllous leaves in *P. euphratica* at a significant SNP (SNP64867 on scaffold NW_011500277.1).

Multi-leaf GWAS for heterophylly detected 200 SNPs that passed permutation tests at the 5% level, among which 151 were associated with PCs (Supplementary Table 3, Figures 5, 6A, and Supplementary Figures 8–15A). Multi-leaf GWAS showed increased power but identified different genomic positions than single-leaf GWAS. Surprisingly, there was only one significantly overlapping SNP (SNP317395) between the single- and multi-leaf GWAS (Supplementary Tables 2, 3). We also found that only one missense SNP (SNP347849) was associated with both the descriptive traits and PCs describing leaf shape. A total of 101 significant SNPs were located in intergenic regions; 5 were missense mutations and 10 were synonymous changes (Supplementary Table 4). The phenotypic variance explained (PVE) by these SNPs ranged from 0.0008 to 9.21% for all traits.

To show how a QTL controls heterophylly, we first compared the descriptive traits of heteromorphic leaves of *P. euphratica* among different genotypes for the significant SNPs, and then evaluated the variation of traits for the three genotypes within the same leaf type. The results presented in Figure 5B and Supplementary Figures 8–10B showed that there were obvious differences between different heteromorphic leaves of the same genotype, with significant QTLs for LL, LW, LI, and LA. SNP25589 was significantly associated with LL in the scaffold NW_011500852.1, and showed differences among the four leaf types (Figure 5C). Comparison among the genotypes of SNP62886 showed differences in LW between linear and lanceolate leaves (Supplementary Figure 8C). A similar result was found for the marker of LA, SNP57868 (Supplementary Figure 10C). For LI, we found that differences among the three genotypes occurred mainly in lanceolate, ovoid, and broad–ovate leaves (Supplementary Figure 9C).

To further show how a QTL controls leaf shape, we examined significant SNPs for each PC. In general, the significant SNPs for PC1 most affected leaf shape variation, as PC1 always explains the most variance. These SNPs were responsible for most of the variance in leaf shape in the ovoid and broad–ovate leaves, in particular. Figure 6A displaying the GWAS result of the PC1 based on the multi-leaf GWAS. Figure 6B shows PC1 for leaf shape for three genotypes, GG, GA, and AA; the QTL was detected by translating EF curves according to leaf shape. Three genotypes of SNP64867 explained the variation of linear and lanceolate leaves according to *x*- and *y*-coordinate values (Figure 6C). Regarding PC2, the shape of heteromorphic leaves associated with genotype GG was markedly different from those of genotypes GC and CC at SNP99561 (Supplementary Figure 11C). Similar results were obtained for the significant markers for PC3 (SNP232072), PC4 (SNP232072), and PC5 (SNP34584) (Supplementary Figures 12–14C). SNP232072 was associated with PC3 and PC4, suggesting that this QTL has a pleiotropic effect on multiple leaf shape features. These results indicate that these QTLs play vital roles in regulating the heterophylly of *P. euphratica.*

### Candidate Genes Underlying the Regulation of Heterophylly

Functional annotation showed that 115 significant SNPs accorded with *P. euphratica* gene models (Supplementary Tables 1, 2). 20 candidate genes exhibited functional associations only with the trait of a single leaf (Table 1), and 29 genes were involved in the heterophylly of *P. euphratica* (Table 2). These genes acted on transcription factors and kinases, and in auxin signaling and cell wall modification. However, there were no overlapping genes between single-and multi-leaf GWAS. Therefore, heterophylly may involve highly specific metabolic pathways or regulatory mechanisms.

**TABLE 1:** Candidate genes identified according to significant SNPs for the different traits based on single-leaf.

Leaf type	Traits	Gene	Variant	Annotation	SNPID
Linear	Leaf width	LOC105112485	Downstream	Transcription factor MYB113	394032
Linear	Leaf index	LOC105115843	Downstream	Ultraviolet-B receptor UVR8	13072
Linear	Leaf index	LOC105122529	Upstream	Auxin response factor 19	24416
Ovoid	Leaf width	LOC105110388	Downstream	MLO-like protein 3	319462
Ovoid	Leaf width	LOC105117940	Upstream	COBRA-like protein 4	56071
Ovoid	Leaf width	LOC105117941	Downstream	COBRA-like protein 1	56075
Ovoid	Leaf width	LOC105113941	Downstream	Transcription factor MYB48	157476
Ovoid	Leaf width	LOC105116215	Downstream	Beta-glucosidase 12	145742, 145744
Ovoid	Leaf width	LOC105115464	Upstream	Uncharacterized LOC105115464	350733, 350735, 350737
Ovoid	Leaf index	LOC105115855	Upstream	Cyclin-T1-3-like	22166
Ovoid	PC2	LOC105131783	Upstream	Stress response protein NST1	89198
Linear	PC1	LOC105110333	Synonymous	Xyloglucan galactosyltransferase KATAMARI1-like	317395
Linear	PC1	LOC105107970	Upstream	Scarecrow-like protein 18	262646
Linear	PC1	LOC105111673	Upstream	Chlorophyll a–b binding protein 151, chloroplastic-like	361059
Lanceolate	PC3	LOC105130018	Missense	Heat stress transcription factor A-6b	75038
Ovoid	PC2	LOC105111343	Synonymous	BRASSINOSTEROID INSENSITIVE 1-associated receptor kinase 1-like	348192
Ovoid	PC3	LOC105111727	Downstream	Protein CHUP1, chloroplastic	364173
Broad-ovate	PC4	LOC105135664	Downstream	Golgi apparatus membrane protein-like protein ECHIDNA	132098
Broad-ovate	PC1	LOC105114785	Non_coding_transcript	LRR receptor-like serine/threonine-protein kinase GSO1	258505
Broad-ovate	PC2	LOC105109333	Upstream	Zeaxanthin epoxidase, chloroplastic-like	292655, 292661

**TABLE 2:** Candidate genes identified according to significant SNPs for the different traits based on multi-leaf GWAS.

Traits	Gene	Variant	Annotation	SNPID
Leaf length	LOC105140600	Downstream	GEM-like protein 5	202558
Leaf length	LOC105116591	Upstream	Peroxidase 55-like	255877
Leaf width	LOC105140604	Upstream	Protein AUXIN SIGNALING F-BOX 2-like	202558
Leaf width	LOC105142145	Downstream	60S acidic ribosomal protein P3-like	231532
Leaf width	LOC105123836	Downstream	Probable starch synthase 4, chloroplastic/amyloplastic	31836
Leaf index	LOC105115441	Missense	Uncharacterized LOC105115441	347844
PC3	LOC105115442	Missense	Uncharacterized LOC105115442	347849
Leaf index	LOC105115442	Synonymous	Uncharacterized LOC105115442	347852
Leaf index	LOC105115442	Synonymous	Uncharacterized LOC105115442	347865
Leaf area	LOC105111467	Downstream	Denticleless protein homolog	352757
Leaf area	LOC105139109	Downstream	Ribosome-binding factor PSRP1, chloroplastic-like	175726
Leaf area	LOC105116653	Downstream	Cytochrome P450 734A1-like	269292
PC1	LOC105115895	Intron	Late embryogenesis abundant protein 1-like	78356
PC2	LOC105113012	Non_coding_transcript	Uncharacterized LOC105113012	58295
PC2	LOC105113012	Synonymous	Uncharacterized LOC105113012	58297
PC2	LOC105113012	Intron	Uncharacterized LOC105113012	58301
PC2	LOC105113012	3_prime_UTR	Uncharacterized LOC105113012	58306
PC2	LOC105113012	Downstream	Uncharacterized LOC105113012	58308 and 58309
PC2, PC3	LOC105115325	Upstream	Putative glycine-rich cell wall structural protein 1	330857 and 330829
PC3	LOC105130289	Intron	Tubulin-folding cofactor C	77148
PC3	LOC105142830	Downstream	Glucan endo-1,3-beta-glucosidase 12	242937
PC3	LOC105115442	Synonymous	Uncharacterized LOC105115442	347840
PC3	LOC105115442	Missense	Uncharacterized LOC105115442	347849
PC4	LOC105133015	Downstream	Pectinesterase/pectinesterase inhibitor 45	101643
PC4	LOC105112781	Upstream	COP9 signalosome complex subunit 3	27366
PC5	LOC105114340	Synonymous	Uncharacterized LOC105114340	199270
PC5	LOC105114340	Non_coding_transcript	Uncharacterized LOC105114340	199282 and 199287
PC5	LOC105111254	Synonymous	Uncharacterized LOC105111254	345110
PC5	LOC105111691	Upstream	Cytochrome P450 94C1-like	362538
PC5	LOC105136555	Downstream	Serine/threonine-protein kinase CDL1-like	143436

Among the candidate genes in the single-leaf GWAS, eight unique genes were closely related to the descriptive traits, while the remaining genes were associated with the PCs. LOC105112485 (transcription factors MYB113 and *MYB113*) and LOC105113941 (transcription factors MYB48 and *MYB48*) were associated with LW in linear and ovoid leaves, respectively (Table 1). LOC105115843 (ultraviolet-B receptor UVR8), which responds to ultraviolet and ultraviolet-B, was associated with LI in ovoid leaves (Table 1). Two genes (LOC105109333 and LOC105122529) were involved in the synthesis and regulation of auxin and ABA, and may play key roles in leaf development. For example, LOC105122529 (auxin response factor 19, *ARF19*), which binds specifically to the DNA sequence 5′-TGTCTC-3′ found in auxin-responsive promoter elements, and which could act as a transcriptional activator or repressor and be functionally redundant with *ARF7*, was associated with LI. Previously, it was reported that rice *ARF19* controls leaf angle through positively regulating *OsGH3-5* and *OsBRI1* (Zhang et al., 2015). In addition, a recent study suggested that *ARF6* and *ARF7* control the flag leaf angle in rice by regulating secondary cell wall biosynthesis of lamina joints (Huang et al., 2021). Based on the function of *ARF19* in rice, we hypothesized that the *P. euphratica* ARF19 protein may similarly regulate leaf development. Several genes, including LOC105135664, LOC105115855, LOC105117940, LOC105117941, and LOC105110388, may control leaf size by regulating cell division and expansion (Table 1).

Multi-leaf GWAS identified 21 genes with SNPs associated with variation in four types of leaves (Table 2). One QTL, associated with PC3 and LI, was located close to the *P. euphratica* LOC105115442 gene mapped on scaffold NW_011500570.1 (Supplementary Table 2), which contained three significant SNPs (SNP347849, missense; SNP347852, synonymous; and SNP347865, synonymous), but this gene was not functionally annotated (Table 2). SNP269292 (associated with LA) and SNP362538 (associated with PC5) were mapped downstream of LOC105116653 (cytochrome P450 734A1-like, *CYP734A1*) and upstream of LOC105111691 (cytochrome P450 94C1-like, *CYP94C1*), respectively (Table 2); these two genes belonged to the same CYP gene family. Chai et al. (2012) reported that the *CYP78A5* gene, a member of the CYN gene family, is involved in regulating leaf development in *Arabidopsis*. This suggests that *CYP734A1* and *CYP94C1* may play important roles in heterophylly. In addition, we identified three genes involved in cell wall organization and modification (LOC105115325, LOC105142830, and LOC105133015) (Table 2), suggesting that cell wall synthesis is associated with photosynthetic performance, which affects leaf outline during the development of *P. euphratica*. Interestingly, we also found that the LOC105113012 gene included five significant SNPs, but the function of this gene remains unknown.

## Discussion

Many plant species exhibit high adaptability, altering leaf morphology in response to environmental perturbations. One of the most remarkable examples of this phenomenon is heterophylly, which is one way of dealing with the spatially and temporally varying environmental conditions presented to a plant throughout its life (Leigh et al., 2011). In some cases, heterophylly plays an important role in ecological and evolutionary processes (Nakayama et al., 2017); however, its genetic signature is still unknown (Nakayama and Kimura, 2015). Although heterophylly has been studied in several species, experimental resources for studying heterophylly in species with typical heteromorphic leaves are lacking (Nakayama et al., 2012). Therefore, a more versatile system to elucidate the mechanisms underlying heterophylly is needed. Existing studies on leaf development have focused on model species, such as *A. thaliana*, rice, and maize (Yang et al., 2011; Zhang et al., 2015; Du et al., 2020). However, these species do not exhibit significant heterophylly, and are therefore not ideal research targets. We used the desert tree *P. euphratica* as a new model species for studying heterophylly, given its marked variation in leaf shape.

*P. euphratica* grows linear, lanceolate, ovoid, and broad–ovate leaves between the juvenile and adult stages, and is therefore a good model species for studying leaf development (Zhao and Qin, 2017). The morphogenesis of *P. euphratica* heterophylly is closely related to its adaptation to high-temperature conditions, salinity, light stimuli, and other stresses (Song et al., 2021). For example, mature *P. euphratica* along river banks do not have more lanceolate leaves than those growing in water-scarce environments. In this study, we comprehensively evaluated the variation of heteromorphic leaves of *P. euphratica* at the population level, based on traditional morphological data and shape information from photographs. The morphologies of the four type leaves were obviously different, indicating heterophylly in this species. One of the highlights of this study was the quantitative description of heterophylly based on GM, which overcomes the inaccuracy of traditional shape analysis depending only on LL, LW, and LA. The shape data from the GM-based approach can precisely capture subtle changes in leaf outline, which is better for evaluating leaf variation among different genotypes of *P. euphratica* (Fu et al., 2013). Orthogonal PCA of “mathematical landmarks” of leaf outline was used to describe the shape changes of leaves. Five PCs explained the variance in heterophylly based on local and global facets of leaf outline.

It is important to obtain information regarding the population structure and genetic relatedness of *P. euphratica* accessions to identify regions of the genome associated with leaf traits. Our population structure analysis suggested that *P. euphratica* genotypes can be divided into two subpopulations accounting for the majority of genotypes. This was somewhat inconsistent with a previous study by Zhang et al. (2020), in which 297 genotypes sampled from the same *P. euphratica* population were classified into six subpopulations, with most individuals in fact originating from just one subpopulation. However, our study sample was relatively large, enabling population stratification with a high degree of confidence. Overall, the structure of our *P. euphratica* population did not exhibit a strong genetic signal, possibly because the genotypes were from a limited area. This may have reduced the false-positive rate in the GWAS.

To explore the genetic mechanism of heterophylly in *P. euphratica* more comprehensively, we used single- and multi-leaf GWAS to identify the genes controlling the formation of heteromorphic leaves. In *P. euphratica*, phenotypic variation may underlie the adaptability of different leaf types to environmental and climate changes. Therefore, the detailed characteristics of different leaf types were integrated into the GWAS framework to solve complex biological questions regarding heterophylly. Multi-leaf GWAS were designed to characterize leaf shape variation in multiple positions on the stem, considering descriptive traits and shape-related PCs through analysis of photographs depicting heterophylly. Linear mixed models have been widely used for association testing, to prevent the inflation associated with false-positives (Li and Zhu, 2013). However, population structure and kinship were not considered in our multi-leaf GWAS, mainly because the single-leaf GWAS demonstrated that including population structure and kinship, as in Q and Q+K models, cannot correct for inflation. Given the complex genetic correlations between multiple traits, mixed models of only one or two traits were used (Korte et al., 2012).

We performed GWAS to examine heterophylly variation based on SNPs obtained by whole-genome resequencing of the largest genome-wide dataset of genetic polymorphisms in *P. euphratica* reported to date. A total of 340 significant SNPs were involved in LL, LW, LI, and LA. There were 140 (41%) associations within 76 genes in the single-leaf GWAS, and 200 (59%) within 101 genes in the multi-leaf GWAS. Approximately half of the SNPs were located in intergenic regions, which may have been because the lack of high-quality complete genome sequences of *P. euphratica* affected the precision of SNP calling. Moreover, intergenic regions are widely distributed across the genome in plants (Yang et al., 2011), which may also be the case for *P. euphratica*. Intergenic areas were previously referred to as “junk DNA” because they were thought to be completely non-functional, lacking even the ability to encode RNA (Rapicavoli et al., 2013). However, some studies suggested that some intergenic regions contain potential open chromatin areas in *Arabidopsis*, rice, and maize, and may act as promoter elements regulating gene expression and quantitative traits (Yang et al., 2011; Du et al., 2020). These intergenic regions may play important roles in *P. euphratica* heterophylly. Despite the high degree of intraspecific variation in descriptive traits and PCs among accessions of *P. euphratica*, we found few variations in the phenotype of lanceolate leaves in the single-leaf GWAS (Supplementary Table 2). This may have been due to the low genomic coverage of the sequence data or to the effects of rare alleles, which are difficult to detect using GWAS.

The single-leaf GWAS identified two MYB transcription factors (*MYB113* and *MYB48*) responsible for changes in leaf shape. Although a small number of MYB genes are involved in leaf development, previous studies focused on model plants and crops. For example, overexpression of *OsMYB103L* in rice was shown to affect the rolled leaf phenotype (Yang et al., 2014). In *A. thaliana*, *MYB113* is associated with the gene *EGL3*, which affects the expression of enzymes involved in later stages of anthocyanin biosynthesis (Mondal and Roy, 2018), and *MYB48*, which regulates flavonol biosynthesis, primarily in cotyledons (Gaudinier et al., 2018). Therefore, *MYB113* and *MYB48* are closely related to the synthesis of flavonoids. Some studies suggested that flavonoid content changes during the development of leaves in various types of plants (Valkama et al., 2004), but the mechanism by which MYB regulates flavonoid synthesis in plants (and thus affects leaf development) is still unknown. Recently, Pan et al. (2020) reported that two types of MYB synergistically regulate transcription during leaf development in the hybrid poplar variety, 84K (*Populus alba* × *Populus glandulosa*). Thus, the *P. euphratica* genes *MYB113* and *MYB48* may play regulatory roles in heterophylly. A significant finding of this study was the identification of two candidate genes involved in hormone biosynthesis, *ARF19* and *ZEP* (LOC105109333). *ARF19* participates in auxin signaling in *Arabidopsis* (Li et al., 2006), and previous studies have shown that it also affects leaf angle in rice (Zhang et al., 2015). *ZEP* plays an important role in the xanthophyll cycle and ABA biosynthesis, and is highly upregulated in salt-stressed *P. euphratica* (Zhang et al., 2019). However, the function of the *ZEP* gene in leaf shape is still unknown.

Our multi-leaf GWAS showed that certain SNPs were associated with the traits of four leaf types. Importantly, the multi-leaf GWAS identified QTLs that control leaf morphology. The *CYP734A1* and *CYP94C1* genes identified in the GWAS have effects on LA. Cytochrome P450 is involved in brassinosteroid (BR) inactivation and regulation of BR homeostasis. *CYP734A1* is associated with *CYP72C1*, inactivating BRs and modulating photomorphogenesis in *Arabidopsis* (Thornton et al., 2010). *CYP94C1*, encoding a bioactive phytohormone that participates in the jasmonate-mediated signaling pathway and plays a role in negative feedback control of jasmonoyl-l-isoleucine levels, also plays a role in the attenuation of jasmonate responses (Kandel et al., 2007). Noir et al. (2013) showed that methyl jasmonate inhibits leaf growth through the jasmonate receptor, COI1, by reducing cell number and size. Therefore, heterophylly appears to be inducible by *CYP94C1*, and its role in *P. euphratica* merits more in-depth analysis. We also identified a potentially pleiotropic QTL (LOC105115442) in *P. euphratica* that was associated with PC3 and LI. This QTL provided novel candidates underlying phenotypic variation, and suggested genomic regions important for heterophylly in *P. euphratica*. *LOC105115441* is a missense gene neighboring *LOC105115442*. Notably, *LOC105115442* was associated with five markers: two missense and three synonymous SNPs. Within *LOC105113012*, five SNPs significantly associated with PC2 were involved in multiple mutation types. This study identified novel candidate genes involved in heterophylly with potential as genetic markers for breeding *P. euphratica*.

## Data Availability Statement

The original contributions presented in the study are publicly available. This data can be found here: https://ngdc.cncb.ac.cn/, GVM000321.

## Author Contributions

XZ and FS performed the data analysis. MS, MY, WB, and AD performed the experiments and conducted fieldwork. RW and XZ conceived of the idea and designed the model. XZ and FS wrote the manuscript. All authors contributed to the article and approved the submitted version.

## Conflict of Interest

The authors declare that the research was conducted in the absence of any commercial or financial relationships that could be construed as a potential conflict of interest.

## Publisher’s Note

All claims expressed in this article are solely those of the authors and do not necessarily represent those of their affiliated organizations, or those of the publisher, the editors and the reviewers. Any product that may be evaluated in this article, or claim that may be made by its manufacturer, is not guaranteed or endorsed by the publisher.
