# Supplementary material for: Genetic Architecture of Heterophylly: Single and Multi-Leaf Genome-Wide Association Mapping in Populus euphratica
Source: Front Plant Sci. 2022 Jun 15;13:870876. doi: 10.3389/fpls.2022.870876 (PMC9240601; doi:10.3389/fpls.2022.870876)
Supplement: Supplementary file 1 [file Table_1.docx]

Table S1. Descriptive statistics and normal distribution for phenotypic traits

| Leaf type | Trait | Mean | SD | CV (%) | Skewness | Kurtosis | *W*-test |
| --- | --- | --- | --- | --- | --- | --- | --- |
| Linear | Leaf length | 6.85 | 2.12 | 30.95 | 0.64 | 4 | <0.01 |
|  | Leaf width | 1.23 | 0.44 | 35.77 | 0.82 | 4.48 | <0.01 |
|  | Leaf index | 5.91 | 1.85 | 31.3 | 1.65 | 6.52 | <0.01 |
|  | Leaf area | 5.86 | 3.4 | 58.02 | 1.07 | 4.25 | <0.01 |
| Lanceolate | Leaf length | 6.19 | 1.78 | 28.71 | 1.23 | 6.14 | <0.01 |
|  | Leaf width | 2.56 | 0.73 | 28.52 | 0.7 | 3.73 | <0.01 |
|  | Leaf index | 2.45 | 0.4 | 16.33 | 1.29 | 4.41 | <0.01 |
|  | Leaf area | 9.88 | 5.51 | 55.77 | 1.84 | 8.83 | <0.01 |
| Ovoid | Leaf length | 4.91 | 1.16 | 23.67 | 0.78 | 4.45 | <0.01 |
|  | Leaf width | 3.81 | 0.94 | 24.74 | 0.53 | 3.6 | <0.01 |
|  | Leaf index | 1.31 | 0.23 | 17.56 | 0.66 | 2.7 | <0.01 |
|  | Leaf area | 11.84 | 5.42 | 45.78 | 1.28 | 5.38 | <0.01 |
| Broad-ovate | Leaf length | 3.67 | 0.91 | 24.8 | 0.54 | 4.18 | <0.01 |
|  | Leaf width | 4.89 | 1.15 | 23.52 | 0.5 | 3.97 | <0.01 |
|  | Leaf index | 0.75 | 0.11 | 14.67 | 0.12 | 2.5 | <0.01 |
|  | Leaf area | 12.78 | 6.31 | 49.37 | 1.57 | 7.91 | <0.01 |
| Linear | PC1 | 853.8 | 356.18 | 41.72 | 0.62 | 2.61 | <0.01 |
|  | PC2 | 452.26 | 142.93 | 31.6 | -0.07 | 3.24 | 0.27 |
|  | PC3 | 350.33 | 106.34 | 30.35 | 0.6 | 2.61 | <0.01 |
|  | PC4 | 231.95 | 46.55 | 20.07 | 1.18 | 6 | <0.01 |
|  | PC5 | 198.3 | 37 | 18.66 | 0.71 | 4.3 | <0.01 |
| Lanceolate | PC1 | 716.46 | 128.97 | 18 | 2.47 | 13.67 | <0.01 |
|  | PC2 | 156.2 | 39.21 | 25.1 | -0.27 | 3.82 | 0.02 |
|  | PC3 | 269.83 | 40.59 | 15.04 | 0.79 | 4.32 | <0.01 |
|  | PC4 | 124.27 | 24.09 | 19.39 | 2.04 | 9.85 | <0.01 |
|  | PC5 | 75.48 | 22.72 | 30.1 | 0.25 | 3.7 | 0.11 |
| Ovoid | PC1 | 233.73 | 64.11 | 27.43 | 0.25 | 3.05 | 0.08 |
|  | PC2 | 108.47 | 30.47 | 28.09 | -0.08 | 2.47 | 0.03 |
|  | PC3 | 95.61 | 23.52 | 24.6 | 1.17 | 4.26 | <0.01 |
|  | PC4 | 133.09 | 33.59 | 25.24 | 0.62 | 3.38 | <0.01 |
|  | PC5 | 97.86 | 21.32 | 21.79 | -0.3 | 4.38 | <0.01 |
| Broad-ovate | PC1 | 181.25 | 43.02 | 23.74 | 0.22 | 2.64 | <0.01 |
|  | PC2 | 249.63 | 21.34 | 8.55 | -0.14 | 3.08 | 0.34 |
|  | PC3 | 47.83 | 18.79 | 39.28 | 1.21 | 4.85 | <0.01 |
|  | PC4 | 121.63 | 36.01 | 29.61 | 0.28 | 2.86 | <0.01 |
|  | PC5 | 59.07 | 17.49 | 29.61 | -0.14 | 2.79 | 0.34 |

Note: SD, standard deviation; CV, coefficient of variation; W-test, Shapiro–Wilk test, if P < 0.05, rejecting the null hypothesis, and there is significant difference between the data and that originating from a normal distribution.

Table S2. Single nucleotide polymorphisms with significant leaf phenotypic associations in 860 *P. euphratica* genotypes based on single-leaf GWAS

| Leaf type | Trait | SNPID | Chr. | Scafflod | Position | Allele | P-value |
| --- | --- | --- | --- | --- | --- | --- | --- |
| Linear | Leaf length | 173641 | 12 | NW_011500187.1 | 237219 | A/G | 4.89e-06 |
| Linear | Leaf length | 173647 | 12 | NW_011500187.1 | 242936 | C/T | 1.07e-05 |
| Linear | Leaf width | 58806 | 3 | NW_011499983.1 | 561267 | C/A | 1.03e-05 |
| Linear | Leaf width | 61857 | 3 | NW_011500691.1 | 47053 | T/C | 1.12e-05 |
| Linear | Leaf width | 115874 | 7 | NW_011499860.1 | 2231130 | A/G | 2.16e-06 |
| Linear | Leaf width | 132934 | 8 | NW_011500061.1 | 171745 | T/C | 9.03e-06 |
| Linear | Leaf width | 297491 | - | NW_011500357.1 | 95365 | G/A | 9.80e-06 |
| Linear | Leaf width | 321099 | - | NW_011500819.1 | 57838 | C/A | 9.27e-06 |
| Linear | Leaf width | 369921 | - | NW_011500963.1 | 56730 | T/C | 1.11e-05 |
| Linear | Leaf width | 370100 | - | NW_011500564.1 | 30274 | A/G | 1.35e-05 |
| Linear | Leaf width | 394032 | - | NW_011501420.1 | 23199 | C/T | 7.55e-06 |
| Linear | Leaf index | 7623 | 1 | NW_011500317.1 | 133806 | C/T | 1.58e-05 |
| Linear | Leaf index | 13072 | 1 | NW_011500181.1 | 20409 | G/C | 5.61e-06 |
| Linear | Leaf index | 24416 | 1 | NW_011500069.1 | 155727 | A/G | 1.29e-05 |
| Linear | Leaf index | 86278 | 5 | NW_011500508.1 | 2220 | C/T | 1.51e-05 |
| Linear | Leaf index | 136159 | 9 | NW_011499847.1 | 1182683 | T/C | 1.35e-05 |
| Linear | Leaf area | 15342 | 1 | NW_011500301.1 | 210341 | G/T | 5.50e-06 |
| Lanceolate | Leaf length | 271196 | - | NW_011500283.1 | 111000 | G/A | 1.21e-05 |
| Lanceolate | Leaf width | 155770 | 11 | NW_011499973.1 | 603805 | A/G | 1.91e-05 |
| Lanceolate | Leaf width | 189543 | 13 | NW_011499899.1 | 310370 | G/A | 3.84e-06 |
| Ovoid | Leaf length | 37217 | 1 | NW_011500403.1 | 7988 | G/A | 7.44e-06 |
| Ovoid | Leaf length | 359906 | - | NW_011500137.1 | 95145 | A/G | 6.55e-06 |
| Ovoid | Leaf width | 56071 | 3 | NW_011499866.1 | 1057845 | C/T | 7.35e-06 |
| Ovoid | Leaf width | 56075 | 3 | NW_011499866.1 | 1057953 | T/C | 6.04e-06 |
| Ovoid | Leaf width | 88221 | 5 | NW_011500234.1 | 124094 | T/C | 9.15e-06 |
| Ovoid | Leaf width | 88222 | 5 | NW_011500234.1 | 124155 | C/T | 9.26e-06 |
| Ovoid | Leaf width | 145647 | 10 | NW_011500176.1 | 34446 | C/T | 5.26e-06 |
| Ovoid | Leaf width | 145718 | 10 | NW_011500176.1 | 39120 | G/A | 4.23e-06 |
| Ovoid | Leaf width | 145742 | 10 | NW_011500176.1 | 48282 | G/A | 6.28e-06 |
| Ovoid | Leaf width | 145744 | 10 | NW_011500176.1 | 48314 | A/C | 5.04e-06 |
| Ovoid | Leaf width | 145752 | 10 | NW_011500176.1 | 64208 | C/T | 6.18e-06 |
| Ovoid | Leaf width | 157348 | 11 | NW_011499885.1 | 300969 | G/C | 6.36e-06 |
| Ovoid | Leaf width | 157359 | 11 | NW_011499885.1 | 309398 | T/G | 5.20e-06 |
| Ovoid | Leaf width | 157445 | 11 | NW_011499885.1 | 415679 | T/A | 3.84e-06 |
| Ovoid | Leaf width | 157476 | 11 | NW_011499885.1 | 506380 | A/G | 6.23e-06 |
| Ovoid | Leaf width | 221089 | 16 | NW_011500143.1 | 311113 | A/G | 2.01e-06 |
| Ovoid | Leaf width | 228555 | 17 | NW_011499948.1 | 573180 | T/A | 4.68e-06 |
| Ovoid | Leaf width | 293233 | - | NW_011501146.1 | 38019 | G/A | 1.53e-06 |
| Ovoid | Leaf width | 293241 | - | NW_011501146.1 | 52511 | C/T | 8.60e-07 |
| Ovoid | Leaf width | 293247 | - | NW_011501146.1 | 52809 | G/A | 8.03e-06 |
| Ovoid | Leaf width | 293259 | - | NW_011500747.1 | 48381 | A/G | 2.12e-06 |
| Ovoid | Leaf width | 293275 | - | NW_011500747.1 | 49002 | G/A | 2.20e-06 |
| Ovoid | Leaf width | 293288 | - | NW_011500747.1 | 51133 | C/T | 5.67e-07 |
| Ovoid | Leaf width | 296024 | - | NW_011501155.1 | 17251 | C/T | 3.40e-06 |
| Ovoid | Leaf width | 296047 | - | NW_011501155.1 | 18180 | C/T | 1.14e-05 |
| Ovoid | Leaf width | 296048 | - | NW_011501155.1 | 18538 | A/T | 8.08e-06 |
| Ovoid | Leaf width | 296118 | - | NW_011501155.1 | 23253 | G/A | 9.88e-06 |
| Ovoid | Leaf width | 296161 | - | NW_011501155.1 | 41262 | G/A | 6.36e-06 |
| Ovoid | Leaf width | 296163 | - | NW_011501155.1 | 41319 | A/G | 9.88e-06 |
| Ovoid | Leaf width | 319462 | - | NW_011500416.1 | 46364 | T/C | 6.24e-07 |
| Ovoid | Leaf width | 350609 | - | NW_011500908.1 | 72523 | T/C | 2.92e-06 |
| Ovoid | Leaf width | 350642 | - | NW_011501308.1 | 25273 | C/T | 7.21e-06 |
| Ovoid | Leaf width | 350678 | - | NW_011501308.1 | 37342 | G/C | 4.08e-06 |
| Ovoid | Leaf width | 350685 | - | NW_011500509.1 | 793 | C/A | 7.58e-06 |
| Ovoid | Leaf width | 350688 | - | NW_011500509.1 | 3934 | T/A | 2.46e-06 |
| Ovoid | Leaf width | 350721 | - | NW_011500509.1 | 18702 | C/T | 8.47e-06 |
| Ovoid | Leaf width | 350733 | - | NW_011500509.1 | 20249 | C/T | 9.35e-07 |
| Ovoid | Leaf width | 350735 | - | NW_011500509.1 | 20634 | G/A | 5.57e-07 |
| Ovoid | Leaf width | 350737 | - | NW_011500509.1 | 20776 | T/C | 1.35e-06 |
| Ovoid | Leaf index | 22166 | 1 | NW_011500054.1 | 440060 | C/T | 4.77e-06 |
| Ovoid | Leaf index | 89198 | 5 | NW_011500226.1 | 148556 | A/G | 4.41e-06 |
| Ovoid | Leaf index | 104438 | 6 | NW_011500158.1 | 173361 | A/G | 9.53e-06 |
| Ovoid | Leaf index | 149507 | 10 | NW_011499979.1 | 453234 | A/C | 7.97e-06 |
| Ovoid | Leaf index | 179049 | 13 | NW_011500627.1 | 17994 | C/T | 5.15e-06 |
| Ovoid | Leaf index | 342922 | - | NW_011500486.1 | 109762 | T/C | 1.70e-06 |
| Ovoid | Leaf index | 342923 | - | NW_011500486.1 | 109856 | A/G | 7.51e-06 |
| Ovoid | Leaf area | 93280 | 5 | NW_011499869.1 | 1303082 | A/G | 6.38e-06 |
| Ovoid | Leaf area | 230059 | 17 | NW_011500773.1 | 34484 | T/C | 1.89e-06 |
| Broad-ovate | Leaf length | 207017 | 15 | NW_011500060.1 | 308546 | G/A | 9.43e-06 |
| Broad-ovate | Leaf length | 290261 | - | NW_011501133.1 | 26643 | A/G | 8.43e-06 |
| Broad-ovate | Leaf width | 8944 | 1 | NW_011499851.1 | 1769532 | T/C | 4.75e-06 |
| Broad-ovate | Leaf width | 164171 | 11 | NW_011500091.1 | 75377 | A/C | 7.33e-06 |
| Broad-ovate | Leaf width | 204567 | 15 | NW_011500023.1 | 277071 | G/A | 3.32e-06 |
| Broad-ovate | Leaf width | 250837 | 19 | NW_011500658.1 | 22159 | T/C | 5.90e-06 |
| Broad-ovate | Leaf width | 355596 | - | NW_011500922.1 | 7825 | G/A | 2.19e-06 |
| Broad-ovate | Leaf index | 19682 | 1 | NW_011500194.1 | 276832 | A/G | 6.77e-06 |
| Broad-ovate | Leaf index | 23989 | 1 | NW_011499995.1 | 270092 | A/G | 1.03e-05 |
| Broad-ovate | Leaf index | 112627 | 6 | NW_011499854.1 | 544034 | T/A | 1.05e-05 |
| Broad-ovate | Leaf index | 112724 | 6 | NW_011499854.1 | 867826 | G/T | 9.31e-06 |
| Broad-ovate | Leaf index | 203160 | 15 | NW_011500191.1 | 213854 | T/C | 3.64e-06 |
| Broad-ovate | Leaf index | 323494 | - | NW_011501228.1 | 16108 | A/G | 9.67e-06 |
| Broad-ovate | Leaf index | 340415 | - | NW_011500879.1 | 58157 | C/T | 9.20e-06 |
| Broad-ovate | Leaf index | 375232 | - | NW_011500976.1 | 52456 | A/T | 1.12e-05 |
| Broad-ovate | Leaf area | 8944 | 1 | NW_011499851.1 | 1769532 | T/C | 1.80e-06 |
| Broad-ovate | Leaf area | 242669 | 18 | NW_011500465.1 | 7835 | G/T | 9.07e-06 |
| Broad-ovate | Leaf area | 266851 | - | NW_011500673.1 | 69622 | A/C | 1.94e-06 |
| Broad-ovate | Leaf area | 266852 | - | NW_011500673.1 | 69677 | A/G | 2.35e-06 |
| Broad-ovate | Leaf area | 355596 | - | NW_011500922.1 | 7825 | G/A | 2.78e-07 |
| Linear | PC1 | 87382 | 5 | NW_011500185.1 | 166155 | A/C | 5.68e-06 |
| Linear | PC1 | 87383 | 5 | NW_011500185.1 | 166175 | A/G | 2.85e-06 |
| Linear | PC1 | 108728 | 6 | NW_011500013.1 | 2896 | C/T | 1.13e-05 |
| Linear | PC1 | 262646 | - | NW_011500660.1 | 22011 | A/G | 1.38e-06 |
| Linear | PC1 | 317395 | - | NW_011500811.1 | 14164 | G/A | 4.58e-06 |
| Linear | PC1 | 361030 | - | NW_011500538.1 | 112403 | G/T | 2.91e-06 |
| Linear | PC1 | 361039 | - | NW_011500538.1 | 120948 | G/A | 8.58e-06 |
| Linear | PC1 | 361059 | - | NW_011500538.1 | 123900 | C/T | 3.32e-06 |
| Linear | PC2 | 82478 | 4 | NW_011500011.1 | 360560 | A/T | 9.43e-06 |
| Linear | PC2 | 314733 | - | NW_011500404.1 | 117778 | T/G | 4.52e-06 |
| Linear | PC3 | 1362 | 1 | NW_011499906.1 | 504979 | A/G | 8.48e-06 |
| Linear | PC4 | 145824 | 10 | NW_011500176.1 | 128526 | C/T | 1.43e-05 |
| Linear | PC4 | 314732 | - | NW_011500404.1 | 117732 | G/A | 1.36e-05 |
| Linear | PC4 | 314733 | - | NW_011500404.1 | 117778 | T/G | 1.16e-06 |
| Lanceolate | PC1 | 293192 | - | NW_011501146.1 | 26322 | C/T | 6.38e-06 |
| Lanceolate | PC2 | 11172 | 1 | NW_011500293.1 | 94882 | C/T | 7.96e-06 |
| Lanceolate | PC2 | 111577 | 6 | NW_011500244.1 | 113285 | A/G | 7.16e-06 |
| Lanceolate | PC3 | 75038 | 4 | NW_011500092.1 | 132607 | T/C | 6.77e-06 |
| Lanceolate | PC3 | 189074 | 13 | NW_011499923.1 | 660205 | A/G | 1.26e-05 |
| Lanceolate | PC3 | 355613 | - | NW_011500922.1 | 26045 | G/C | 1.95e-05 |
| Lanceolate | PC4 | 95114 | 5 | NW_011500037.1 | 445032 | G/A | 9.51e-06 |
| Lanceolate | PC4 | 192232 | 13 | NW_011499978.1 | 549575 | G/A | 3.98e-06 |
| Lanceolate | PC4 | 246550 | 19 | NW_011500102.1 | 233102 | G/A | 6.35e-07 |
| Lanceolate | PC5 | 161520 | 11 | NW_011500026.1 | 96109 | C/T | 6.58e-06 |
| Lanceolate | PC5 | 185313 | 13 | NW_011499862.1 | 2175776 | C/T | 7.71e-06 |
| Ovoid | PC2 | 89198 | 5 | NW_011500226.1 | 148556 | A/G | 1.28e-05 |
| Ovoid | PC2 | 171571 | 12 | NW_011499865.1 | 202639 | T/G | 5.53e-06 |
| Ovoid | PC2 | 321453 | - | NW_011500420.1 | 129610 | G/A | 5.22e-06 |
| Ovoid | PC2 | 348192 | - | NW_011500502.1 | 68473 | C/T | 8.65e-06 |
| Ovoid | PC3 | 364173 | - | NW_011501349.1 | 44546 | C/T | 6.11e-06 |
| Ovoid | PC5 | 339560 | - | NW_011501275.1 | 35535 | C/T | 2.09e-06 |
| Ovoid | PC5 | 339561 | - | NW_011501275.1 | 35536 | G/A | 5.21e-06 |
| Ovoid | PC5 | 392478 | - | NW_011500219.1 | 195372 | T/C | 1.02e-05 |
| Broad-ovate | PC1 | 90533 | 5 | NW_011499967.1 | 283814 | C/T | 7.78e-06 |
| Broad-ovate | PC1 | 132098 | 8 | NW_011500732.1 | 77463 | C/T | 4.91e-06 |
| Broad-ovate | PC1 | 258505 | - | NW_011500649.1 | 22042 | A/G | 4.05e-06 |
| Broad-ovate | PC1 | 348263 | - | NW_011500502.1 | 124720 | G/A | 7.38e-06 |
| Broad-ovate | PC2 | 132590 | 8 | NW_011500249.1 | 203975 | A/T | 8.33e-06 |
| Broad-ovate | PC2 | 292644 | - | NW_011501145.1 | 24592 | C/T | 3.23e-06 |
| Broad-ovate | PC2 | 292655 | - | NW_011501145.1 | 25225 | A/G | 5.14e-06 |
| Broad-ovate | PC2 | 292661 | - | NW_011501145.1 | 25438 | A/T | 1.31e-06 |
| Broad-ovate | PC2 | 292691 | - | NW_011501145.1 | 31901 | A/G | 3.00e-06 |
| Broad-ovate | PC2 | 336700 | - | NW_011500869.1 | 77781 | G/A | 8.22e-06 |
| Broad-ovate | PC3 | 344540 | - | NW_011500891.1 | 54728 | G/A | 1.12e-05 |
| Broad-ovate | PC4 | 90533 | 5 | NW_011499967.1 | 283814 | C/T | 5.01e-06 |
| Broad-ovate | PC4 | 104331 | 6 | NW_011500158.1 | 84232 | A/G | 8.65e-06 |
| Broad-ovate | PC4 | 132098 | 8 | NW_011500732.1 | 77463 | C/T | 1.22e-05 |
| Broad-ovate | PC4 | 230810 | 17 | NW_011500516.1 | 21691 | G/A | 9.29e-06 |
| Broad-ovate | PC4 | 258505 | - | NW_011500649.1 | 22042 | A/G | 1.01e-06 |
| Broad-ovate | PC4 | 388748 | - | NW_011500609.1 | 55813 | A/C | 1.21e-05 |
| Broad-ovate | PC5 | 41976 | 2 | NW_011499849.1 | 3594063 | T/A | 3.61e-06 |
| Broad-ovate | PC5 | 312410 | - | NW_011501197.1 | 2369 | T/A | 8.67e-06 |

Table S3. Single nucleotide polymorphisms with significant leaf phenotypic associations in 860 *P. euphratica* genotypes based on multi-leaf GWAS

| Trait | SNPID | Chr. | Scafflod | Position | Allele | P-value |
| --- | --- | --- | --- | --- | --- | --- |
| Leaf length | 58783 | Chr03 | NW_011499983.1 | 560087 | C/T | 2.82e-05 |
| Leaf length | 154497 | Chr11 | NW_011500803.1 | 79720 | G/A | 1.27e-05 |
| Leaf length | 202558 | Chr15 | NW_011499946.1 | 737585 | C/T | 2.88e-05 |
| Leaf length | 255565 | Chr19 | NW_011500077.1 | 181950 | C/T | 3.05e-05 |
| Leaf length | 255877 | Chr19 | NW_011500045.1 | 135153 | C/T | 8.85e-06 |
| Leaf length | 274189 | - | NW_011500290.1 | 84297 | A/G | 2.34e-05 |
| Leaf length | 330641 | - | NW_011500450.1 | 110777 | A/G | 1.20e-06 |
| Leaf length | 399713 | - | NW_011500640.1 | 30457 | T/G | 3.03e-05 |
| Leaf width | 31836 | Chr01 | NW_011499900.1 | 748911 | T/G | 2.76e-05 |
| Leaf width | 62886 | Chr03 | NW_011500082.1 | 400538 | A/G | 3.87e-05 |
| Leaf width | 138950 | Chr09 | NW_011500490.1 | 35790 | G/A | 2.95e-05 |
| Leaf width | 168170 | Chr12 | NW_011499938.1 | 744781 | G/A | 3.46e-05 |
| Leaf width | 202558 | Chr15 | NW_011499946.1 | 737585 | C/T | 2.00e-05 |
| Leaf width | 204697 | Chr15 | NW_011500023.1 | 544423 | C/T | 5.00e-05 |
| Leaf width | 219378 | Chr16 | NW_011499936.1 | 728664 | C/T | 3.12e-05 |
| Leaf width | 219453 | Chr16 | NW_011499936.1 | 848565 | T/G | 2.94e-05 |
| Leaf width | 221176 | Chr16 | NW_011500143.1 | 326550 | A/G | 4.92e-05 |
| Leaf width | 231532 | Chr17 | NW_011500382.1 | 84830 | C/T | 4.86e-05 |
| Leaf width | 258621 | - | NW_011500649.1 | 47168 | A/G | 2.36e-05 |
| Leaf width | 290036 | - | NW_011501130.1 | 52418 | C/T | 1.94e-05 |
| Leaf width | 290073 | - | NW_011501130.1 | 57130 | A/G | 1.25e-05 |
| Leaf width | 347136 | - | NW_011500899.1 | 55139 | G/A | 3.30e-05 |
| Leaf index | 76846 | Chr04 | NW_011499848.1 | 2980133 | T/C | 7.46e-05 |
| Leaf index | 88734 | Chr05 | NW_011500686.1 | 57458 | G/A | 3.54e-05 |
| Leaf index | 116758 | Chr07 | NW_011499870.1 | 693966 | A/G | 4.92e-06 |
| Leaf index | 144964 | Chr10 | NW_011500107.1 | 22602 | C/T | 3.76e-05 |
| Leaf index | 308566 | - | NW_011500384.1 | 147892 | A/T | 5.34e-05 |
| Leaf index | 347844 | - | NW_011500501.1 | 37565 | G/A | 9.00e-06 |
| Leaf index | 347849 | - | NW_011500501.1 | 39214 | C/T | 3.48e-05 |
| Leaf index | 347852 | - | NW_011500501.1 | 39578 | T/G | 1.25e-05 |
| Leaf index | 347865 | - | NW_011500501.1 | 41484 | G/C | 6.50e-05 |
| Leaf index | 361030 | - | NW_011500538.1 | 112403 | G/T | 4.68e-05 |
| Leaf index | 361035 | - | NW_011500538.1 | 120843 | C/T | 2.41e-05 |
| Leaf index | 365388 | - | NW_011500953.1 | 10202 | C/T | 1.10e-05 |
| Leaf index | 397873 | - | NW_011500638.1 | 57926 | C/T | 5.50e-05 |
| Leaf area | 52339 | Chr03 | NW_011499859.1 | 1886558 | C/T | 2.06e-05 |
| Leaf area | 57868 | Chr03 | NW_011500171.1 | 210012 | C/T | 3.48e-05 |
| Leaf area | 114029 | Chr07 | NW_011500053.1 | 52624 | C/T | 3.05e-05 |
| Leaf area | 123098 | Chr07 | NW_011500259.1 | 212735 | G/A | 1.88e-05 |
| Leaf area | 128217 | Chr08 | NW_011499845.1 | 2968972 | A/G | 2.32e-05 |
| Leaf area | 157597 | Chr11 | NW_011499885.1 | 694369 | A/C | 2.89e-05 |
| Leaf area | 173734 | Chr12 | NW_011501037.1 | 3310 | T/C | 3.43e-05 |
| Leaf area | 175726 | Chr12 | NW_011500457.1 | 51590 | C/T | 4.27e-05 |
| Leaf area | 253832 | Chr19 | NW_011499933.1 | 485671 | C/T | 2.29e-05 |
| Leaf area | 255528 | Chr19 | NW_011500077.1 | 138293 | T/G | 1.82e-05 |
| Leaf area | 269292 | - | NW_011501476.1 | 13655 | A/G | 3.86e-05 |
| Leaf area | 278364 | - | NW_011500700.1 | 1108 | T/A | 3.86e-05 |
| Leaf area | 329930 | - | NW_011500448.1 | 86893 | A/G | 4.63e-05 |
| Leaf area | 348734 | - | NW_011500503.1 | 34168 | A/T | 3.63e-05 |
| Leaf area | 352757 | - | NW_011501314.1 | 37471 | A/C | 3.04e-05 |
| Leaf area | 393221 | - | NW_011501419.1 | 11312 | G/A | 2.58e-05 |
| PC1 | 36001 | Chr01 | NW_011500476.1 | 51009 | T/C | 1.29e-04 |
| PC1 | 56006 | Chr03 | NW_011499866.1 | 1039315 | G/C | 8.20e-05 |
| PC1 | 60384 | Chr03 | NW_011500066.1 | 223218 | C/A | 1.27e-04 |
| PC1 | 64846 | Chr04 | NW_011500599.1 | 34960 | G/C | 4.14e-05 |
| PC1 | 64867 | Chr04 | NW_011500599.1 | 43511 | G/A | 8.69e-05 |
| PC1 | 66948 | Chr04 | NW_011500574.1 | 71543 | C/T | 8.10e-05 |
| PC1 | 72900 | Chr04 | NW_011499918.1 | 660769 | A/T | 7.54e-05 |
| PC1 | 73334 | Chr04 | NW_011499918.1 | 932913 | G/A | 9.33e-05 |
| PC1 | 77875 | Chr04 | NW_011499915.1 | 32753 | G/A | 1.30e-04 |
| PC1 | 82879 | Chr04 | NW_011499970.1 | 285503 | G/A | 5.24e-05 |
| PC1 | 112983 | Chr06 | NW_011499854.1 | 1316820 | T/G | 1.18e-04 |
| PC1 | 114883 | Chr07 | NW_011499860.1 | 505380 | T/C | 4.80e-05 |
| PC1 | 115744 | Chr07 | NW_011499860.1 | 2097058 | G/A | 1.21e-04 |
| PC1 | 146498 | Chr10 | NW_011499872.1 | 1323816 | G/A | 1.26e-04 |
| PC1 | 161366 | Chr11 | NW_011500254.1 | 133297 | G/A | 6.30e-05 |
| PC1 | 162859 | Chr11 | NW_011499997.1 | 401973 | A/T | 1.40e-04 |
| PC1 | 167091 | Chr12 | NW_011499976.1 | 611945 | A/C | 1.44e-04 |
| PC1 | 177726 | Chr12 | NW_011500434.1 | 134373 | C/T | 1.13e-04 |
| PC1 | 203616 | Chr15 | NW_011499863.1 | 254953 | A/G | 1.15e-04 |
| PC1 | 208312 | Chr15 | NW_011501099.1 | 57640 | T/G | 8.69e-05 |
| PC1 | 211305 | Chr15 | NW_011500225.1 | 83868 | G/A | 1.30e-04 |
| PC1 | 232072 | Chr17 | NW_011500086.1 | 335694 | C/A | 8.36e-05 |
| PC1 | 243837 | Chr19 | NW_011500168.1 | 54419 | C/T | 1.43e-05 |
| PC1 | 247329 | Chr19 | NW_011500003.1 | 393664 | G/A | 6.70e-05 |
| PC1 | 261707 | - | NW_011501057.1 | 28453 | C/T | 3.29e-05 |
| PC1 | 262443 | - | NW_011500659.1 | 105092 | G/A | 6.00e-05 |
| PC1 | 262444 | - | NW_011500659.1 | 105118 | G/A | 5.82e-05 |
| PC1 | 307682 | - | NW_011501181.1 | 46080 | G/A | 3.58e-05 |
| PC1 | 327819 | - | NW_011500843.1 | 39589 | A/G | 1.01e-04 |
| PC1 | 329838 | - | NW_011500448.1 | 63885 | C/T | 9.72e-05 |
| PC1 | 340016 | - | NW_011500878.1 | 13924 | C/T | 4.53e-05 |
| PC1 | 372848 | - | NW_011500969.1 | 63755 | T/C | 3.79e-05 |
| PC1 | 384778 | - | NW_011501000.1 | 14144 | A/G | 4.48e-05 |
| PC2 | 240 | Chr01 | NW_011500021.1 | 423841 | A/T | 7.66e-05 |
| PC2 | 25474 | Chr01 | NW_011499987.1 | 254082 | A/T | 3.26e-05 |
| PC2 | 39779 | Chr01 | NW_011500579.1 | 18851 | C/T | 3.27e-05 |
| PC2 | 41641 | Chr02 | NW_011499849.1 | 1542024 | C/T | 7.29e-05 |
| PC2 | 58295 | Chr03 | NW_011499983.1 | 226989 | A/C | 4.37e-05 |
| PC2 | 58297 | Chr03 | NW_011499983.1 | 227947 | C/T | 4.37e-05 |
| PC2 | 58301 | Chr03 | NW_011499983.1 | 228767 | G/A | 3.26e-05 |
| PC2 | 58306 | Chr03 | NW_011499983.1 | 232404 | A/G | 4.37e-05 |
| PC2 | 58308 | Chr03 | NW_011499983.1 | 256432 | G/A | 7.50e-05 |
| PC2 | 58309 | Chr03 | NW_011499983.1 | 256435 | A/C | 7.50e-05 |
| PC2 | 58352 | Chr03 | NW_011499983.1 | 355373 | T/C | 1.40e-05 |
| PC2 | 63592 | Chr03 | NW_011500067.1 | 3342 | T/C | 6.30e-05 |
| PC2 | 83684 | Chr04 | NW_011500230.1 | 61489 | C/T | 1.78e-05 |
| PC2 | 99561 | Chr05 | NW_011499846.1 | 4872284 | C/G | 1.54e-05 |
| PC2 | 191629 | Chr13 | NW_011500397.1 | 146087 | C/T | 4.02e-05 |
| PC2 | 198655 | Chr14 | NW_011500588.1 | 51277 | A/G | 3.55e-05 |
| PC2 | 236449 | Chr18 | NW_011499850.1 | 1180495 | A/G | 1.18e-05 |
| PC2 | 237008 | Chr18 | NW_011499850.1 | 2960022 | C/T | 5.08e-05 |
| PC2 | 238829 | Chr18 | NW_011500540.1 | 64702 | G/T | 2.39e-05 |
| PC2 | 243051 | Chr18 | NW_011500371.1 | 134520 | A/G | 6.99e-05 |
| PC2 | 262437 | - | NW_011500659.1 | 100452 | C/T | 5.63e-05 |
| PC2 | 262439 | - | NW_011500659.1 | 100554 | G/A | 4.47e-05 |
| PC2 | 262443 | - | NW_011500659.1 | 105092 | G/A | 1.85e-05 |
| PC2 | 262444 | - | NW_011500659.1 | 105118 | G/A | 2.59e-05 |
| PC2 | 262445 | - | NW_011500659.1 | 105126 | T/C | 6.70e-05 |
| PC2 | 262446 | - | NW_011500659.1 | 105128 | C/T | 7.98e-05 |
| PC2 | 281930 | - | NW_011501110.1 | 4850 | C/T | 7.27e-05 |
| PC2 | 281933 | - | NW_011501110.1 | 5179 | A/G | 3.88e-05 |
| PC2 | 285073 | - | NW_011500716.1 | 77050 | A/G | 1.17e-05 |
| PC2 | 294415 | - | NW_011500751.1 | 1684 | G/A | 4.93e-05 |
| PC2 | 330857 | - | NW_011500851.1 | 13527 | G/A | 4.79e-05 |
| PC2 | 334755 | - | NW_011500062.1 | 27952 | G/A | 3.13e-05 |
| PC2 | 350921 | - | NW_011500509.1 | 83467 | G/T | 8.04e-05 |
| PC2 | 358724 | - | NW_011500535.1 | 42735 | C/T | 4.77e-05 |
| PC2 | 369918 | - | NW_011500963.1 | 55479 | C/A | 5.92e-05 |
| PC3 | 9079 | Chr01 | NW_011499851.1 | 2626645 | T/G | 5.98e-05 |
| PC3 | 66948 | Chr04 | NW_011500574.1 | 71543 | C/T | 8.11e-05 |
| PC3 | 77148 | Chr04 | NW_011499848.1 | 4658555 | A/G | 3.33e-05 |
| PC3 | 148451 | Chr10 | NW_011500598.1 | 76899 | T/C | 1.10e-04 |
| PC3 | 161469 | Chr11 | NW_011500026.1 | 25464 | C/T | 1.05e-04 |
| PC3 | 212751 | Chr16 | NW_011500129.1 | 202570 | A/T | 2.33e-05 |
| PC3 | 232072 | Chr17 | NW_011500086.1 | 335694 | C/A | 5.63e-05 |
| PC3 | 242937 | Chr18 | NW_011500371.1 | 8286 | C/T | 5.54e-05 |
| PC3 | 243837 | Chr19 | NW_011500168.1 | 54419 | C/T | 3.93e-05 |
| PC3 | 262444 | - | NW_011500659.1 | 105118 | G/A | 7.29e-05 |
| PC3 | 281048 | - | NW_011501506.1 | 24249 | A/T | 6.11e-05 |
| PC3 | 311125 | - | NW_011500392.1 | 56837 | A/C | 8.91e-05 |
| PC3 | 315392 | - | NW_011501205.1 | 32636 | C/A | 1.09e-04 |
| PC3 | 325563 | - | NW_011501235.1 | 3173 | G/A | 2.63e-05 |
| PC3 | 330829 | - | NW_011500851.1 | 11452 | T/C | 3.86e-05 |
| PC3 | 336369 | - | NW_011500869.1 | 29331 | C/T | 6.18e-05 |
| PC3 | 347840 | - | NW_011500501.1 | 37231 | C/T | 6.20e-05 |
| PC3 | 347849 | - | NW_011500501.1 | 39214 | C/T | 7.35e-05 |
| PC3 | 361708 | - | NW_011500943.1 | 40691 | C/G | 5.82e-05 |
| PC3 | 377256 | - | NW_011500582.1 | 75811 | G/A | 6.10e-05 |
| PC4 | 27364 | Chr01 | NW_011500016.1 | 470532 | T/G | 5.21e-05 |
| PC4 | 27366 | Chr01 | NW_011500016.1 | 504362 | T/C | 4.45e-05 |
| PC4 | 27442 | Chr01 | NW_011500282.1 | 36128 | C/T | 4.66e-05 |
| PC4 | 27955 | Chr01 | NW_011500205.1 | 162273 | T/C | 4.75e-05 |
| PC4 | 37862 | Chr01 | NW_011499920.1 | 109008 | A/G | 5.64e-05 |
| PC4 | 40746 | Chr01 | NW_011499871.1 | 890119 | T/A | 6.35e-05 |
| PC4 | 64846 | Chr04 | NW_011500599.1 | 34960 | G/C | 1.46e-05 |
| PC4 | 64867 | Chr04 | NW_011500599.1 | 43511 | G/A | 1.76e-05 |
| PC4 | 67816 | Chr04 | NW_011499904.1 | 711542 | T/A | 6.18e-05 |
| PC4 | 67822 | Chr04 | NW_011499904.1 | 751809 | T/C | 4.73e-05 |
| PC4 | 101643 | Chr06 | NW_011499852.1 | 622682 | A/G | 5.99e-05 |
| PC4 | 114883 | Chr07 | NW_011499860.1 | 505380 | T/C | 1.81e-05 |
| PC4 | 148451 | Chr10 | NW_011500598.1 | 76899 | T/C | 3.93e-05 |
| PC4 | 188309 | Chr13 | NW_011499856.1 | 3054066 | A/C | 1.65e-05 |
| PC4 | 193034 | Chr13 | NW_011500614.1 | 24609 | C/T | 2.59e-05 |
| PC4 | 206044 | Chr15 | NW_011499905.1 | 1168947 | C/T | 3.16e-05 |
| PC4 | 207383 | Chr15 | NW_011500438.1 | 132903 | T/G | 5.95e-05 |
| PC4 | 207384 | Chr15 | NW_011500438.1 | 132968 | T/C | 4.41e-06 |
| PC4 | 207385 | Chr15 | NW_011500438.1 | 132969 | G/T | 8.67e-06 |
| PC4 | 207393 | Chr15 | NW_011500438.1 | 133398 | G/T | 4.51e-05 |
| PC4 | 232072 | Chr17 | NW_011500086.1 | 335694 | C/A | 4.99e-06 |
| PC4 | 235014 | Chr18 | NW_011500320.1 | 145091 | G/T | 1.01e-05 |
| PC4 | 243837 | Chr19 | NW_011500168.1 | 54419 | C/T | 2.36e-05 |
| PC4 | 280970 | - | NW_011501106.1 | 9141 | C/T | 6.41e-05 |
| PC4 | 311125 | - | NW_011500392.1 | 56837 | A/C | 5.61e-05 |
| PC4 | 346219 | - | NW_011501297.1 | 37985 | G/A | 4.04e-05 |
| PC4 | 356450 | - | NW_011500525.1 | 69266 | T/G | 2.35e-05 |
| PC5 | 81366 | Chr04 | NW_011500192.1 | 35947 | C/T | 1.22e-04 |
| PC5 | 127497 | Chr08 | NW_011499845.1 | 2312512 | A/T | 1.20e-04 |
| PC5 | 143436 | Chr09 | NW_011500402.1 | 67433 | A/C | 1.40e-05 |
| PC5 | 161371 | Chr11 | NW_011500254.1 | 133441 | C/T | 1.18e-04 |
| PC5 | 177462 | Chr12 | NW_011501084.1 | 15757 | C/T | 9.84e-05 |
| PC5 | 177471 | Chr12 | NW_011501084.1 | 32470 | A/G | 1.36e-04 |
| PC5 | 189266 | Chr13 | NW_011499923.1 | 979613 | G/A | 7.56e-05 |
| PC5 | 199208 | Chr14 | NW_011500539.1 | 63204 | T/A | 7.12e-05 |
| PC5 | 199240 | Chr14 | NW_011500539.1 | 76553 | A/G | 1.29e-04 |
| PC5 | 199270 | Chr14 | NW_011500539.1 | 110132 | T/C | 4.86e-05 |
| PC5 | 199282 | Chr14 | NW_011500828.1 | 5743 | C/G | 1.27e-05 |
| PC5 | 199287 | Chr14 | NW_011500828.1 | 8369 | A/G | 5.18e-06 |
| PC5 | 208677 | Chr15 | NW_011500670.1 | 51842 | C/T | 1.01e-04 |
| PC5 | 212054 | Chr16 | NW_011500345.1 | 43385 | A/G | 1.24e-04 |
| PC5 | 215639 | Chr16 | NW_011500064.1 | 124615 | A/G | 1.19e-04 |
| PC5 | 228106 | Chr17 | NW_011499948.1 | 77432 | A/G | 1.26e-04 |
| PC5 | 236053 | Chr18 | NW_011499850.1 | 509239 | G/C | 2.14e-06 |
| PC5 | 242968 | Chr18 | NW_011500371.1 | 75961 | T/G | 1.63e-05 |
| PC5 | 243329 | Chr19 | NW_011500281.1 | 62785 | C/A | 1.29e-04 |
| PC5 | 252331 | Chr19 | NW_011500056.1 | 190723 | G/A | 7.81e-05 |
| PC5 | 261428 | - | NW_011501455.1 | 42214 | A/G | 1.06e-04 |
| PC5 | 263533 | - | NW_011500663.1 | 12123 | G/C | 1.31e-04 |
| PC5 | 268300 | - | NW_011501074.1 | 55453 | A/G | 8.82e-05 |
| PC5 | 289138 | - | NW_011501126.1 | 18695 | C/T | 5.98e-05 |
| PC5 | 296377 | - | NW_011500756.1 | 13161 | T/G | 3.71e-05 |
| PC5 | 303853 | - | NW_011500769.1 | 49811 | G/T | 3.59e-05 |
| PC5 | 333896 | - | NW_011501260.1 | 3224 | A/G | 4.52e-05 |
| PC5 | 339666 | - | NW_011500876.1 | 64191 | C/T | 5.90e-05 |
| PC5 | 345110 | - | NW_011500494.1 | 2533 | A/G | 1.23e-04 |
| PC5 | 345841 | - | NW_011501295.1 | 46092 | G/A | 2.90e-05 |
| PC5 | 362370 | - | NW_011501345.1 | 38673 | A/C | 2.07e-05 |
| PC5 | 362538 | - | NW_011500547.1 | 1256 | G/A | 1.36e-04 |
| PC5 | 362573 | - | NW_011500547.1 | 10102 | T/A | 8.81e-05 |
| PC5 | 395016 | - | NW_011501026.1 | 33326 | T/C | 1.06e-04 |

Table S4 Candidate genes identified according to significant SNPs for the different traits based on single-leaf GWAS

| Gene | Variant  by | Annotation | GO | SNPID |
| --- | --- | --- | --- | --- |
| LOC105139948 | 3_prime_UTR | mediator of RNA polymerase II transcription subunit 15a-like | BP: positive regulation of fatty acid biosynthetic,  response to salicylic acid | 58806 |
| LOC105124642 | non_coding_transcript | agamous-like MADS-box protein AGL62 | MF: DNA-binding transcription factor activity; BP: regulation of auxin polar transport | 115874 |
| LOC105114025 | downstream | probable splicing factor 3A subunit 1 | MF: RNA binding; BP: mRNA splicing, via spliceosome | 297491 |
| LOC105116474 | upstream | peroxidase 6 | - | 321099 |
| LOC105108362 | downstream | hexokinase-1-like | MF: kinase activity | 24416 |
| LOC105140070 | downstream | Putative MATE efflux family protein 3, chloroplastic-like | MF: antiporter activity | 136159 |
| LOC105130112 | downstream | probable glutathione S-transferase | MF: glutathione transferase activity; BP: protein glutathionylation | 157445 |
| LOC105130132 | downstream | heat shock 70 kDa protein 16-like | MF: ATPase activity | 157476 |
| LOC105114540 | upstream | beta-glucosidase 12-like | MF: beta-glucosidase activity; BP: response to salt stress | 319462 |
| LOC105126873 | non_coding_transcript | protein trichome birefringence-like 10 | MF: xylan O-acetyltransferase activity; BP: [cellulose biosynthetic process](https://www.ebi.ac.uk/QuickGO/term/GO:0030244); [pectin biosynthetic process](https://www.ebi.ac.uk/QuickGO/term/GO:0045489); [plant-type cell wall modification](https://www.ebi.ac.uk/QuickGO/term/GO:0009827); [xylan biosynthetic process](https://www.ebi.ac.uk/QuickGO/term/GO:0045492) | 93280 |
| LOC105108043 | non_coding_transcript | probable S-adenosylmethionine-dependent methyltransferase At5g38100 | MF: S-adenosylmethionine-dependent methyltransferase activity; BP: methylation | 207017 |
| LOC105121520 | downstream | inactive poly [ADP-ribose] polymerase RCD1 | MF: NAD+ ADP-ribosyltransferase activity; BP: embryo development ending in seed dormancy, ethylene-activated signaling pathway, jasmonic acid mediated signalin pathway,nitric oxide biosynthetic process,  programmed cell death,  regulation of reactive oxygen species metabolic process,  response to ethylene, response to osmotic stress,  response to ozone, response to salt stress, response to superoxide deprivation | 8944 |
| LOC105109133 | intron | GDSL esterase/lipase At1g29670-like | MF: hydrolase activity, acting on ester bonds | 164171 |
| LOC105115891 | non_coding_transcript | probable magnesium transporter NIPA6 | MF: protein self-association; BP: magnesium ion transport | 250837 |
| LOC105122482 | downstream | CAAX prenyl protease 2 | MF: endopeptidase actvity, metalloendopeptidase activity, peptidase activity; BP: CAAX-box protein processing, proteolysis | 112627 |
| LOC105116693 | intron | eukaryotic peptide chain release factor GTP-binding subunit ERF3A-like | MF: GTPase activity,  GTP binding, translation release factor activity; BP: cytoplasmic translational termination, translation | 340415 |
| LOC105114861 | upstream | glucan endo-1,3-beta-glucosidase 13 | MF: cellulase activity  glucan endo-1,3-beta-D-glucosidase activity; BP: systemic acquired resistance | 242669 |
| LOC105115931 | intron | probable sugar phosphate/phosphate translocator At3g17430 | MF: antiporter activity; BP:  carbohydrate transport | 266851, 266852 |
| LOC105141463 | downstream | 39S ribosomal protein L4, mitochondrial | - | 82478 |
| LOC105133395 | intron | phosphatidylserine decarboxylase proenzyme 2-like | MF: phosphatidylserine decarboxylase activity; BP:  phospholipid biosynthetic process | 314733 |
| LOC105111441 | intron | probable isoleucine--tRNA ligase, mitochondrial | MF: isoleucine-tRNA ligase activity; BP: isoleucyl-tRNA aminoacylation | 1362 |
| LOC105113284 | intron | COBW domain-containing protein 1-like | - | 11172 |
| LOC105135383 | downstream | putative DNA-binding protein ESCAROLA | MF: minor groove of adenine-thymine-rich DNA binding; BP: protein transport | 189074 |
| LOC105116826 | non_coding_transcript | protease Do-like 8, chloroplastic | MF: peptidase activity,  serine-type endopeptidase activity; BP: photosystem II repair, proteolysis | 355613 |
| LOC105142819 | downstream | nifU-like protein 3, chloroplastic | MF: iron ion binding,  structural molecule activity; BP: chloroplast organization,  iron-sulfur cluster assembly | 95114 |
| LOC105139645 | upstream | tetraketide alpha-pyrone reductase 1-like | BP: pollen development, pollen exine formation, seed development, sporopollenin biosynthetic process | 192232 |
| LOC105109507 | downstream | ketol-acid reductoisomerase, chloroplastic-like | MF: isomerase activity;  oxidoreductase activity; BP:  branched-chain amino acid biosynthetic process Source: InterPro | 246550 |
| LOC105125236 | upstream | germin-like protein 5-1 | MF: manganese ion binding; BP: plasmodesmata-mediated intercellular transport; regulation of root development | 185313 |
| LOC105116182 | non_coding_transcript | 5'-nucleotidase domain-containing protein 4-like | MF: 5'-nucleotidase activity,  metal ion binding | 132098 |
| LOC105111139 | upstream | xaa-Pro dipeptidase | MF: metal ion binding,  metallopeptidase activity,  proline dipeptidase activity | 104331 |
| LOC105116182 | intron | 5'-nucleotidase domain-containing protein 4-like | MF: 5'-nucleotidase activity, metal ion binding | 132098 |
| LOC105120711 | upstream | actin-related protein 2/3 complex subunit 5A-like | - | 41976 |

Table S5 Candidate genes identified according to significant SNPs for the different traits based on multi-leaf GWAS

| Gene | Variant  by | Annotation | GO | SNPID |
| --- | --- | --- | --- | --- |
| LOC105139943 | upstream | mediator of RNA polymerase II transcription subunit 15a-like | MF: chromatin DNA binding, transcription coregulator activity | 58783 |
| LOC105137613 | missense | probable serine/threonine protein kinase IRE | MF: kinase activity, metal ion binding, protein serine/threonine kinase activity; BP: intracellular signal transduction, peptidyl-serine phosphorylation, regulation of growth | 202558, 202558 |
| LOC105114765 | upstream | probable polygalacturonase At1g80170 | MF: polygalacturonase activity; BP: carbohydrate metabolic process,  cell wall organization | 330641 |
| LOC105136739 | missense | probable serine/threonine-protein kinase WNK3 | MF: kinase activity, metal ion binding, protein serine/threonine kinase activity; BP: intracellular signal transduction, peptidyl-serine phosphorylation, regulation of growth | 168170 |
| LOC105136542 | upstream | cytochrome P450 716B1-like | MF: heme binding, iron ion binding, monooxygenase activity, oxidoreductase activity; BP: sterol metabolic process | 219453 |
| LOC105115221 | upstream | peptidyl-prolyl cis-trans isomerase CYP21-4-like | MF: isomerase activity | 76846 |
| LOC105127063 | intron | acyl-protein thioesterase 2-like | MF: zinc ion binding | 116758 |
| LOC105111356 | intron | copper-transporting ATPase PAA1, chloroplastic-like | MF: ATPase-coupled cation transmembrane transporter activity, ATP binding,  metal ion binding | 57868 |
| LOC105120518 | downstream | importin-4-like | MF: heme binding,  metal ion binding,  oxygen carrier activity,  small GTPase binding; BP: protein import into nucleus | 128217 |
| LOC105130156 | upstream | septum-promoting GTP-binding protein 1-like | MF: GTPase activity,  GTP binding | 157597 |
| LOC105136237 | missense | small RNA 2'-O-methyltransferase-like | MF: O-methyltransferase activity, RNA binding,  RNA methyltransferase activity; BP: piRNA metabolic process,  production of siRNA involved in RNA interference | 253832 |
| LOC105108681 | upstream | aminoacyl tRNA synthase complex-interacting multifunctional protein 1 | MF: aminoacyl-tRNA ligase activity, molecular adaptor activity; BP: apoptotic process, negative regulation of cell population proliferation, positive regulation of aminoacyl-tRNA ligase activity, positive regulation of protein ubiquitination, protein-containing complex assembly, type II pneumocyte differentiation | 255528 |
| LOC105108250 | intron | protein ROOT HAIR DEFECTIVE 3-like | MF: GTPase activity,  GTP binding; BP: endoplasmic reticulum membrane fusion | 60384 |
| LOC105115590 | intron | DNA-directed RNA polymerase 2B, chloroplastic/mitochondrial-like | - | 64846 |
| LOC105115591 | splice_region | kanadaptin | MF: mRNA | 64867 |
| LOC105115463 | intron | pescadillo homolog | MF: ribonucleoprotein complex binding, RNA binding; BP: cell population proliferation, maturation of 5.8S rRNA from tricistronic rRNA transcript, regulation of cell cycle, ribosomal large subunit biogenesis | 66948 |
| LOC105134834 | synonymous | beta-glucosidase 46-like | MF: beta-glucosidase activity; BP: carbohydrate metabolic process | 72900 |
| LOC105134872 | missense | probable LRR receptor-like serine/threonine-protein kinase At4g20940 | MF: ATP binding, protein kinase activity | 73334 |
| LOC105139216 | upstream | protein SET DOMAIN GROUP 40 |  | 82879 |
| LOC105122561 | 5_prime_UTR | putative multidrug resistance protein | MF: transmembrane transporter activity; BP:  transmembrane transport | 112983 |
| LOC105124468 | intron | calmodulin-binding transcription activator 3-like | MF: calmodulin binding,  double-stranded DNA binding, transcription coregulator activity; BP:  regulation of transcription by RNA polymerase II | 114883 |
| LOC105124621 | missense | endogenous alpha-amylase/subtilisin inhibitor-like | MF: endopeptidase inhibitor activity | 115744 |
| LOC105127592 | intron | probable ribose-5-phosphate isomerase 3, chloroplastic | MF: ribose-5-phosphate isomerase activity; BP:  defense response to bacterium, pentose-phosphate shunt, non-oxidative branch, reductive pentose-phosphate cycle | 146498 |
| LOC105112361 | 3_prime_UTR | histone-lysine N-methyltransferase ATX5-like | MF: metal ion binding,  methyltransferase activity; BP: methylation | 211305 |
| LOC105108983 | upstream, | probable receptor-like protein kinase At1g67000 | MF: ATP binding,  polysaccharide binding,  protein serine kinase activity, protein threonine kinase activity | 232072, |
| LOC105111280 | missense | endoglucanase 9 | MF: cellulase activity,  BP: cellulose catabolic process, cell wall organization | 243837 |
| LOC105141906 | upstream | COP9 signalosome complex subunit 7-like | MF: COP9 signalosome assembly | 240 |
| LOC105139921 | synonymous | uncharacterized protein | - | 58295,  58297,58301 |
| LOC105139922 | synonymous | glycerol kinase | BP: glycerol-3-phosphate biosynthetic process, glycerol metabolic process, phosphorylation, triglyceride metabolic process | 58306 |
| LOC105139925 | synonymous | purple acid phosphatase 22-like | MF: acid phosphatase activity | 58308,  58309 |
| LOC105139934 | intron | topless-related protein 1 | MF: acid phosphatase activity | 58352 |
| LOC105115523 | intron | 3-hydroxyisobutyryl-CoA hydrolase 1-like | MF: 3-hydroxyisobutyryl-CoA hydrolase activity; BP: valine catabolic process | 198655 |
| LOC105120972 | intron | probable serine/threonine-protein kinase Cx32, chloroplastic | MF: ATP binding,  protein serine/threonine kinase activity | 236449 |
| LOC105121322 | upstream | probable LRR receptor-like serine/threonine-protein kinase At1g07560 | MF: ATP binding,  protein serine kinase activity,  protein threonine kinase activity | 237008 |
| LOC105115286 | downstream | nuclear pore complex protein NUP85-like | - | 238829 |
| LOC105114146 | splice_region | vacuolar protein sorting-associated protein 32 homolog 2-like | BP: endosome transport via multivesicular body sorting pathway, intralumenal vesicle formation, late endosome to vacuole transport, protein transport | 243051 |
| LOC105115250 | upstream | GPI transamidase component PIG-T-like | MF: attachment of GPI anchor to protein  Complete GO annotation on QuickG | 358724 |
| LOC105115463 | intron | pescadillo homolog | MF: ribonucleoprotein complex binding; BP: gamete generation, maturation of LSU-rRNA from tricistronic rRNA transcript (SSU-rRNA, 5.8S rRNA, LSU-rRNA), regulation of cell division | 66948 |
| LOC105117829 | 5_prime_UTR | protein CHROMATIN REMODELING 4-like | MF: helicase activity, metal ion binding, nucleosome-dependent; BP: chromatin organization, DNA repair, maintenance of rDNA, pollen development, regulation of histone exchange | 77148 |
| LOC105115585 | synonymous | putative E3 ubiquitin-protein ligase RF298 | MF: metal ion binding, transferase activity; BP: protein ubiquitination | 148451 |
| LOC105110343 | upstream | protein NRT1/ PTR FAMILY 8.1-like | MF: transmembrane transporter activity; BP:  oligopeptide transport,  phosphate ion transport | 212751 |
| LOC105114135 | downstream | acyl-protein thioesterase 2-like | - | 242937 |
| LOC105117976 | downstream | xylosyltransferase 2-like | MF: transferase activity, transferring glycosyl groups; BP: cell wall organization | 281048 |
| LOC105114348 | downstream | senescence-specific cysteine protease SAG39-like | MF: cysteine-type endopeptidase activity; BP:  proteolysis involved in cellular protein catabolic process | 311125 |
| LOC105141709 | non_coding_transcript | probable ATP-dependent DNA helicase HFM1 | MF: ATP binding, DNA helicase activity; BP:  resolution of meiotic recombination intermediates | 27364 |
| LOC105141712 | non_coding_transcript | polyribonucleotide nucleotidyltransferase 2, mitochondrial | MF: 3'-5'-exoribonuclease activity, polyribonucleotide nucleotidyltransferase activity, RNA binding; BP:  mitochondrial mRNA catabolic process | 27366 |
| LOC105113161 | downstream | CAS1 domain-containing protein 1-like | MF: transferase activity | 27442 |
| LOC105135024 | missense | ubiquitin carboxyl-terminal hydrolase 9-like | MF: cysteine-type peptidase activity, thiol-dependent ubiquitin-specific protease activity; BP: protein deubiquitination;  ubiquitin-dependent protein catabolic process | 37862 |
| LOC105115590 | splice_region | DNA-directed RNA polymerase 2B, chloroplastic/mitochondrial-like | - | 64846, 64867 |
| LOC105133246 | intron | peroxisomal (S)-2-hydroxy-acid oxidase-like | MF: acyl-CoA oxidase activity, fatty acid binding,  flavin adenine dinucleotide binding; BP: fatty acid beta-oxidation using acyl-CoA oxidase, lipid homeostasis | 67822 |
| LOC105121743 | upstream | WD repeat-containing protein 3 | MF: flower development,  histone H3-K36 methylation,  histone H3-K4 methylation,  negative regulation of flower development, positive regulation of wax biosynthetic,  posttranscriptional gene silencing | 101643 |
| LOC105124468 | intron | calmodulin-binding transcription activator 3-like | MF: 1-acylglycerol-3-phosphate O-acyltransferase activity, calmodulin binding,  DNA binding, phospholipase A2 activity, phospholipase A2 activity, sterol esterase activity, triglyceride lipase activity | 114883 |
| LOC105123523 | missense | aluminum-activated malate transporter 2-like | BP: malate transport | 188309 |
| LOC105113588,  LOC105115204 | intron | GDSL esterase/lipase At5g45670-like | BP: hydrolase activity, acting on ester bonds | 235014 |
| LOC105117257 | downstream | probable disease resistance protein At4g27220 | MF: ADP binding,  ATP binding; defense response | 280970 |
| LOC105112511 | intron | endoribonuclease Dicer homolog 2-like | MF: ATP binding, DNA binding, helicase activity,  ribonuclease III activity, RNA binding; BP:  production of siRNA involved in RNA | 127497 |
| LOC105117212 | 3_prime_UTR | NAP1-related protein 2-like | MF: chromatin binding, histone binding; BP:  nucleosome assembly | 177471 |
| LOC105115284 | intron | succinyl-CoA ligase [ADP-forming] subunit beta, mitochondrial | MF: ATP binding, magnesium ion binding,  succinate-CoA ligase (ADP-forming) activity; BP: succinyl-CoA metabolic process, succinyl-CoA pathway, tricarboxylic acid cycle | 199270 |
| LOC105115921 | downstream | late secretory pathway protein AVL9-like | - | 208677 |
| LOC105108151 | intron | EH domain-containing protein 1-like | BP: cellular response to nerve growth factor stimulus | 215639 |
| LOC105120907 | downstream | cytochrome P450 CYP73A100-like | MF: heme binding, iron ion binding, monooxygenase activity, oxidoreductase activity, acting on paired donors, with incorporation or reduction of molecular oxygen | 236053 |
| LOC105114140 | upstream | probable caffeoyl-CoA O-methyltransferase At4g26220 | MF: caffeoyl-CoA O-methyltransferase activity,  metal ion binding,  S-adenosylmethionine-dependent methyltransferase activity; BP: lignin biosynthetic process,  methylation | 242968 |
| LOC105116686 | upstream | probable beta-D-xylosidase 5 | MF: alpha-L-arabinofuranosidase activity,  xylan 1,4-beta-xylosidase activity; BP: arabinan catabolic process, xylan catabolic process | 339666 |
