# Supplementary material for: Genetic Architecture of Heterophylly: Single and Multi-Leaf Genome-Wide Association Mapping in Populus euphratica
Source: Front Plant Sci. 2022 Jun 15;13:870876. doi: 10.3389/fpls.2022.870876 (PMC9240601; doi:10.3389/fpls.2022.870876)
Supplement: Supplementary file 2 [file Data_Sheet_1.docx]

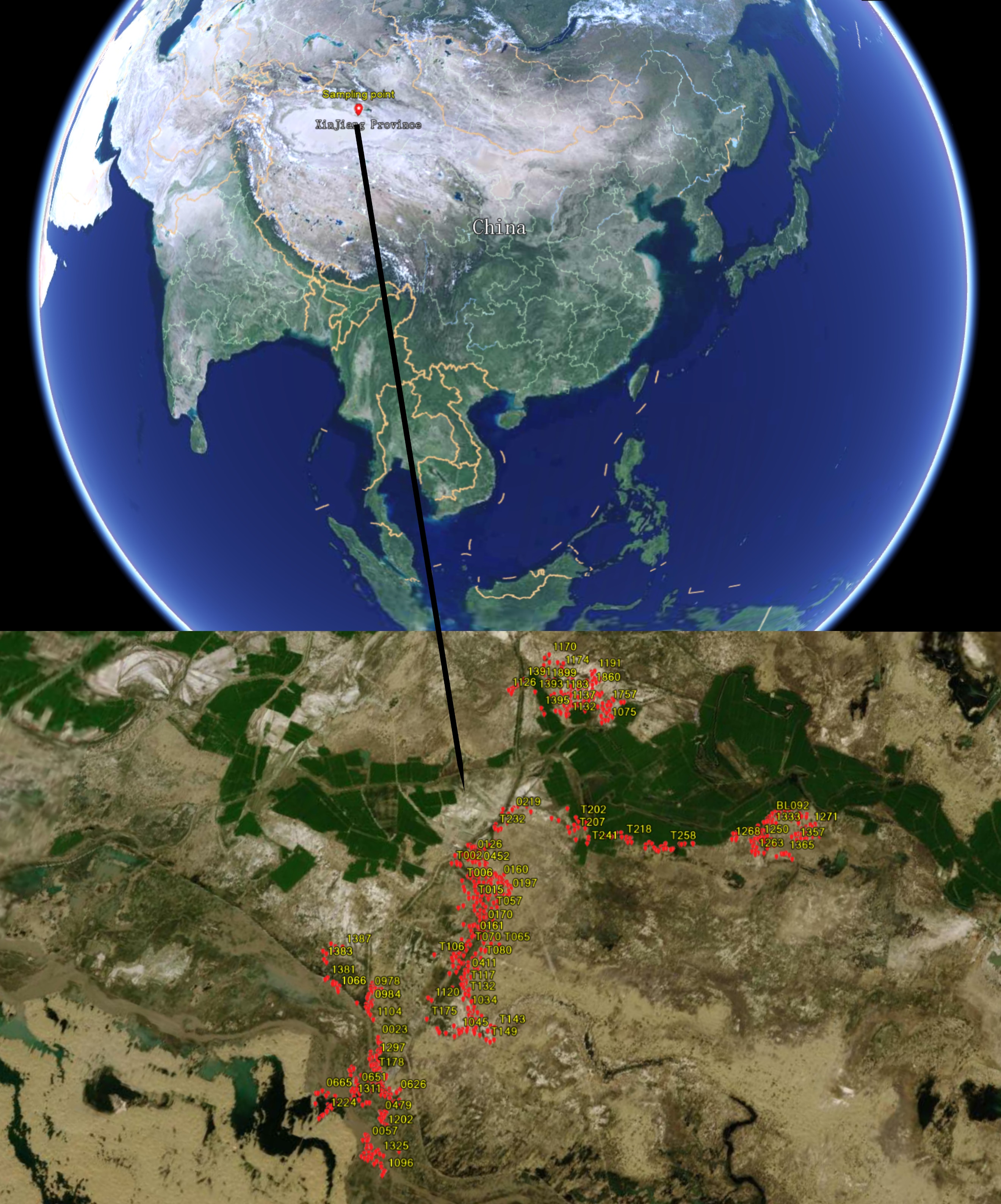


**Figure S1** Geographic distribution of *P. euphuatica* genotypes sampled in this study.


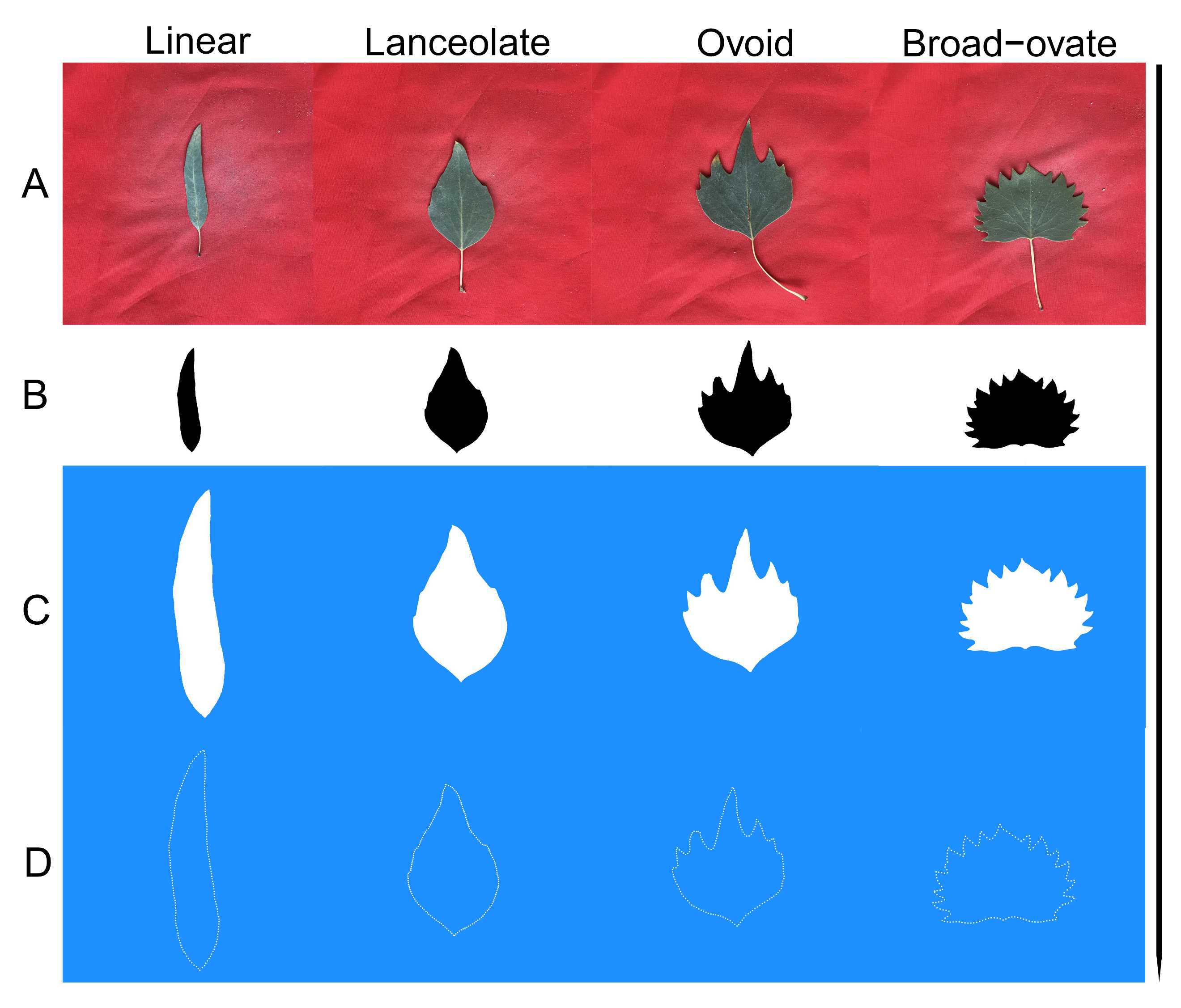


**Figure S2** A workflow of extracting outline information from heterophylly image. (**A**) The raw color images of heterophylly. (**B**) The color image is converted to a black and white binary image. (**C**) The rotation, position and scale effects of heterophylly are removed by shape alignment. (**D**) A set of points on the heterophylly outline spaced at an equal radial angle.


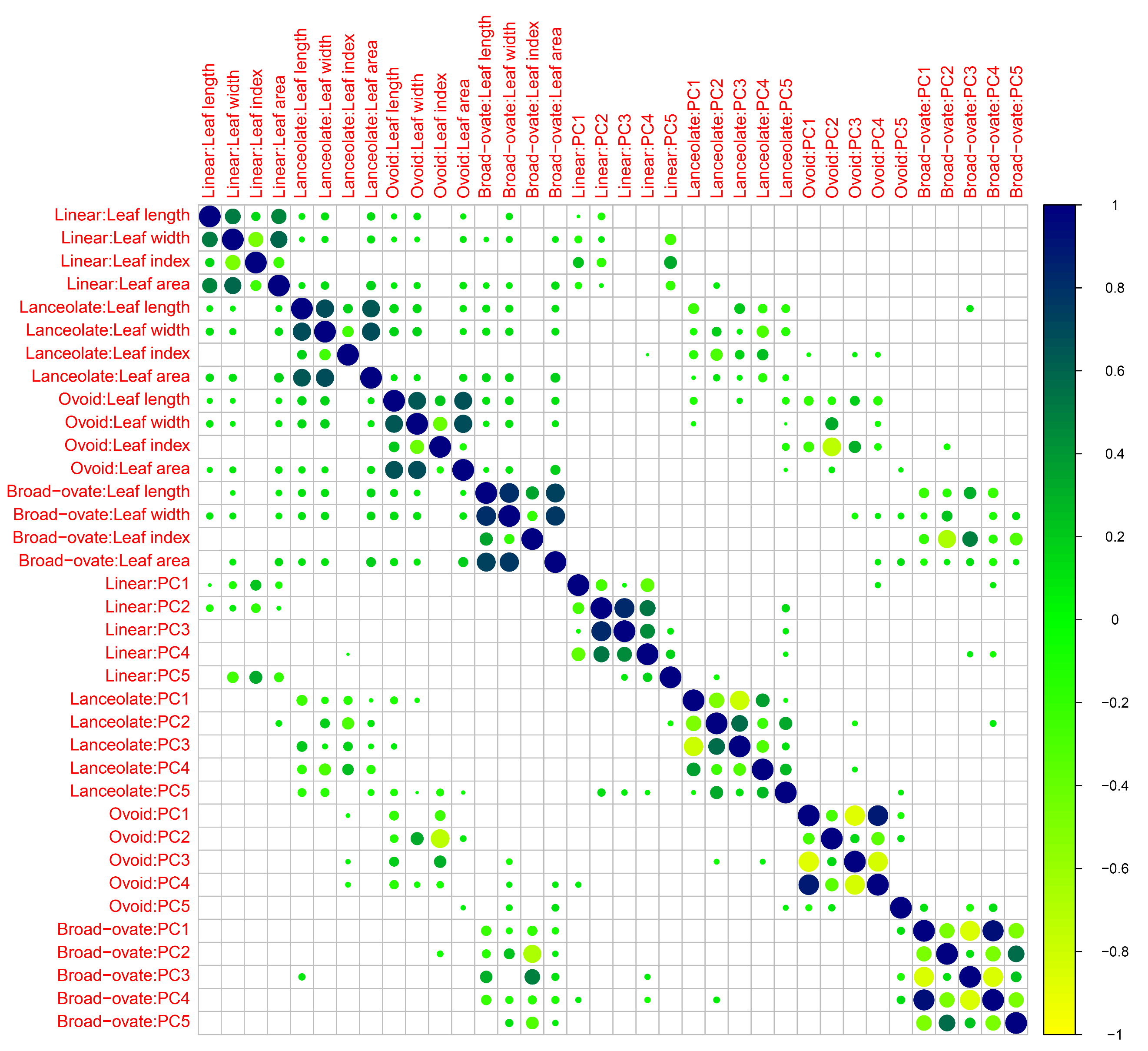


**Figure S3** Phenotypic correlations among all traits include the descriptive phenotypes and the first five PCs of shape for four heterophyllous leaves. The blank squares indicate that there is no significant correlation (P<0.05).


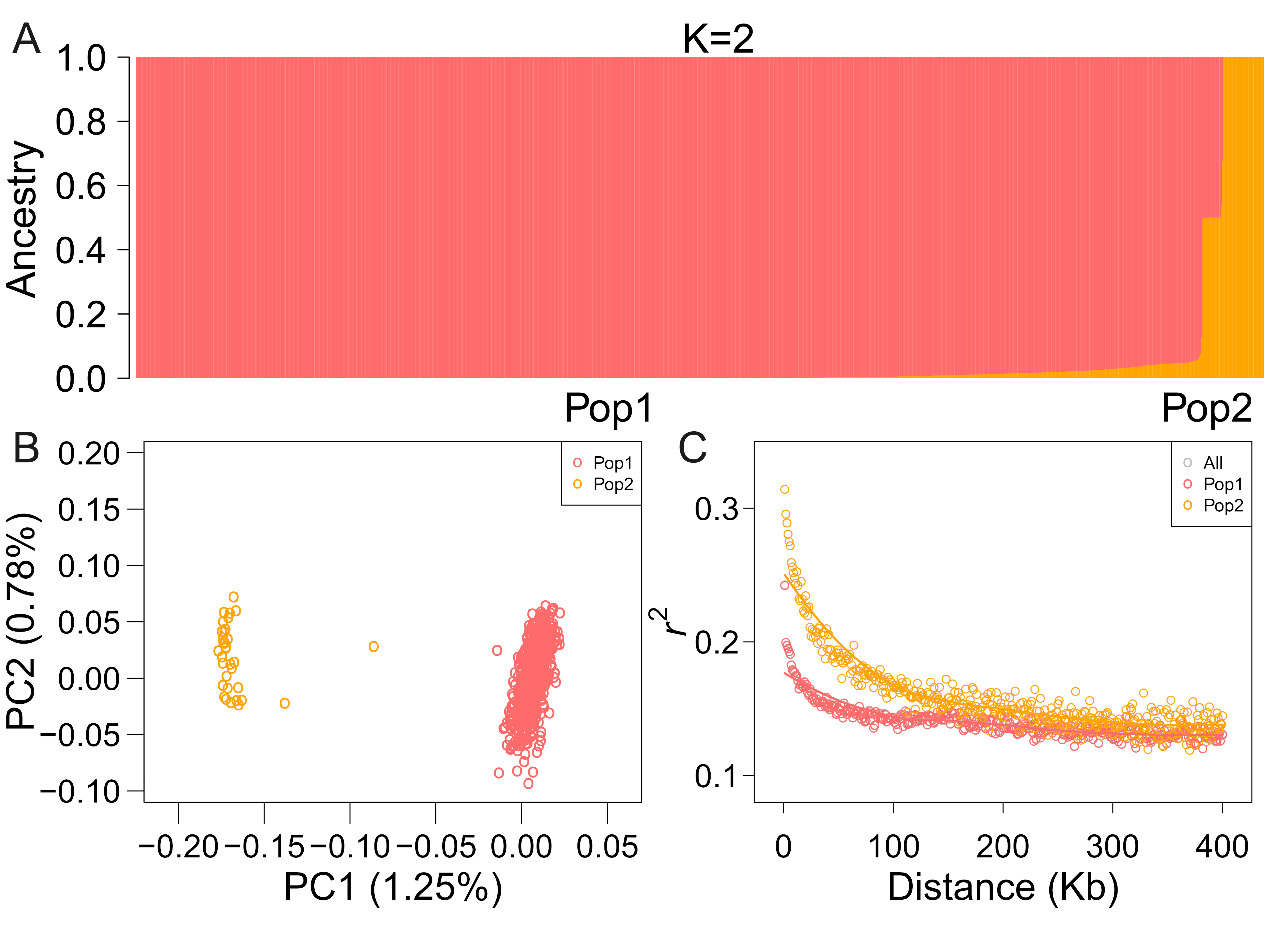


**Figure S4** Population structure and linkage disequilibrium in *P. euphratica*. (**A**) Distruct plot of faststructurefor two subpopulations, where each color represents a subpopulation (Pop1 and Pop2). (**B**) First two principal components. (**C**) Genome-wide linkage disequilibrium decay estimated of the whole population and subpopulations.


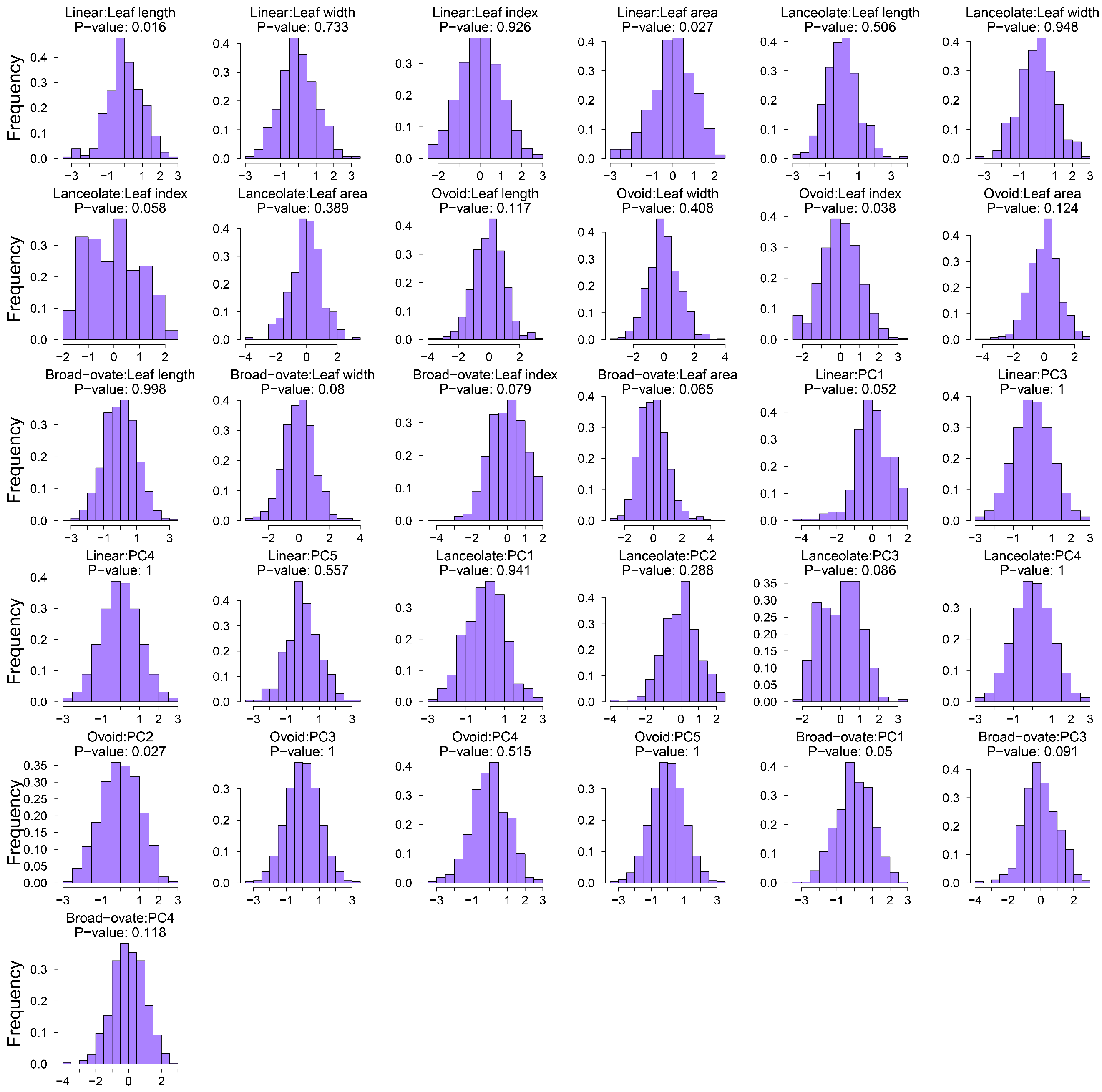


**Figure S5** A histogram of transformed phenotypic whose original data do not follow a normal distribution. *P*-value was calculated by shapiro-wilk test.


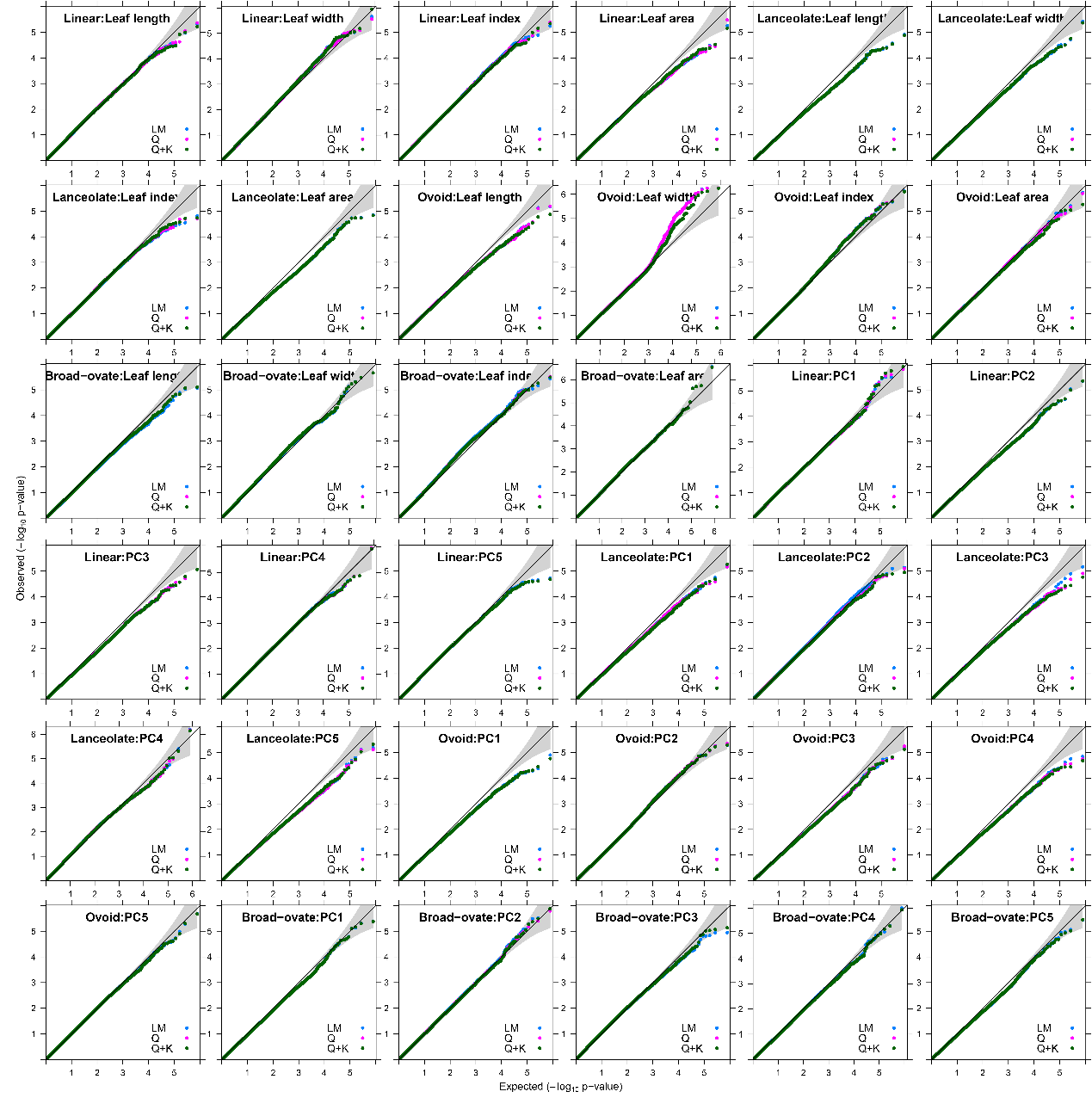


**Figure S6** Quantile-quantile plots of the leaf traits from the single-leaf GWAS scan based on three models.


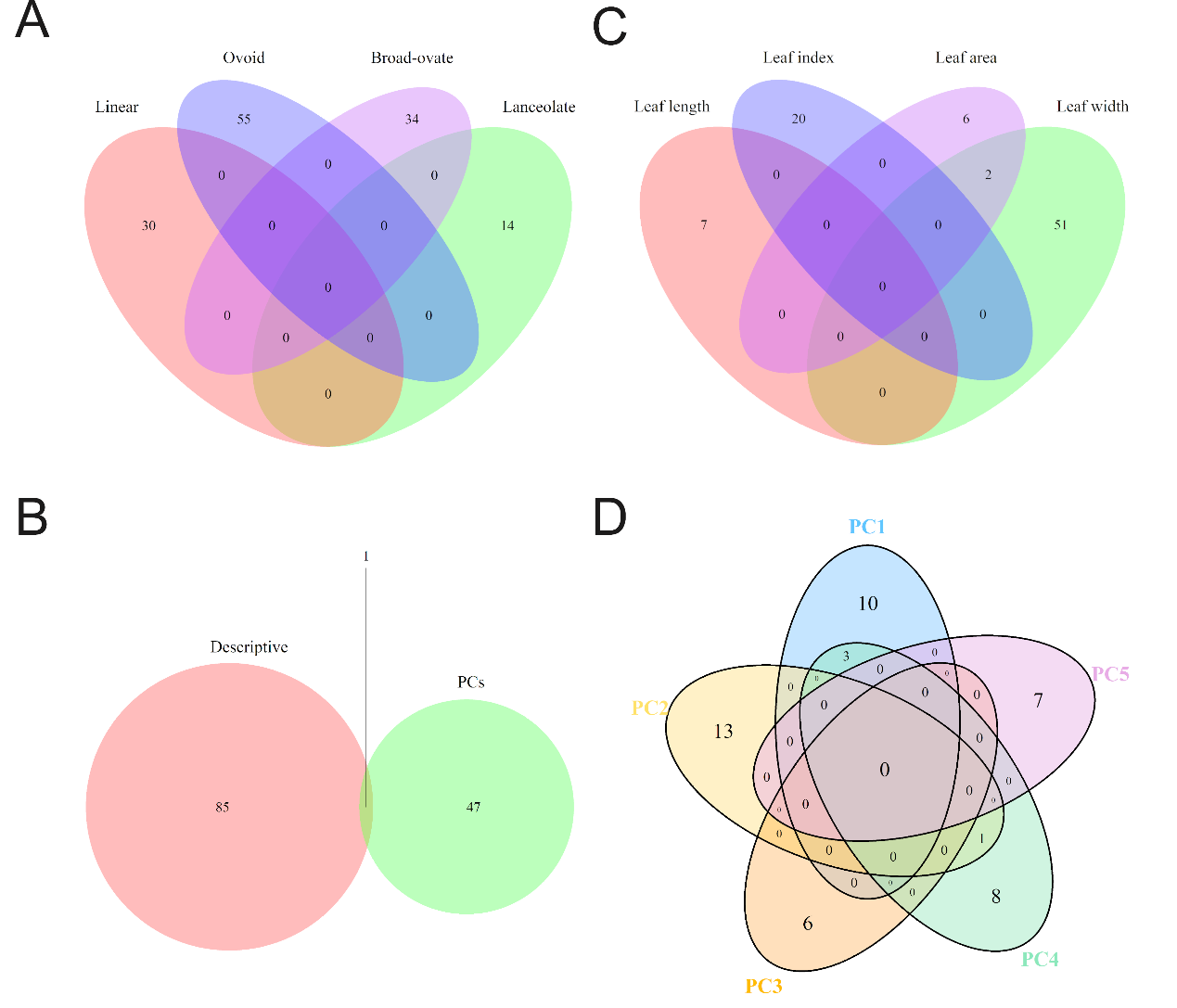


**Figure S7** Venn diagram showing the number of overlapped significant SNPs among the heteromorphic leaves (A), the descriptive traits and PCs (B), the four descriptive traits (C) and the five PCs (D).


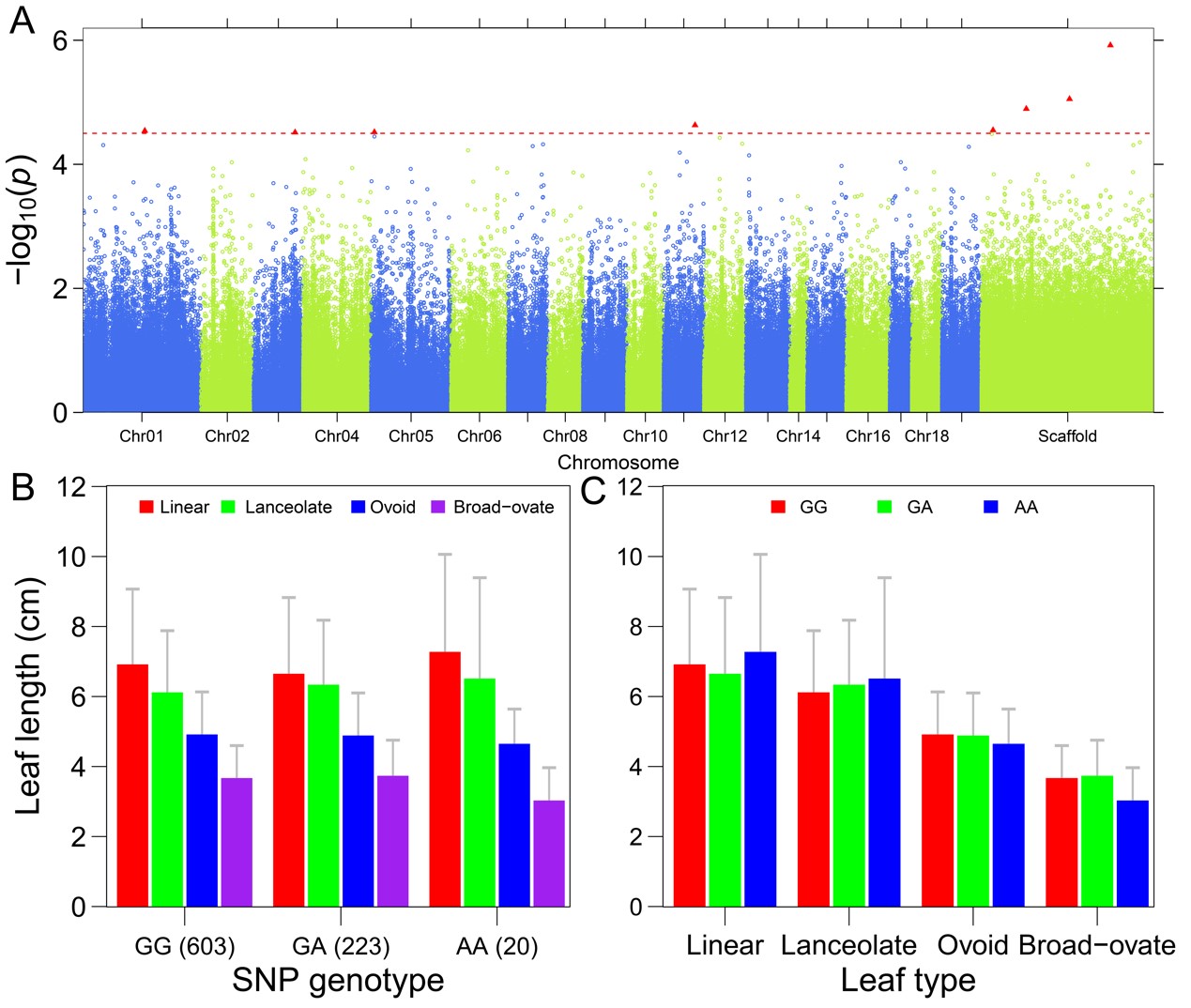


**Figure S8** Genome-wide association mapping of the leaf length in *P. euphratica*. (A) Manhattan plot displaying the GWAS result of the trait ‘leaf length’ based on the single-leaf GWAS. The significantly associated SNP markers are labeled. (B) Histograms of leaf length of different leaf types plotted as a function of genotypes at SNP62886. (C) Histograms of allelic effects of SNP62886 for the heterophylly.


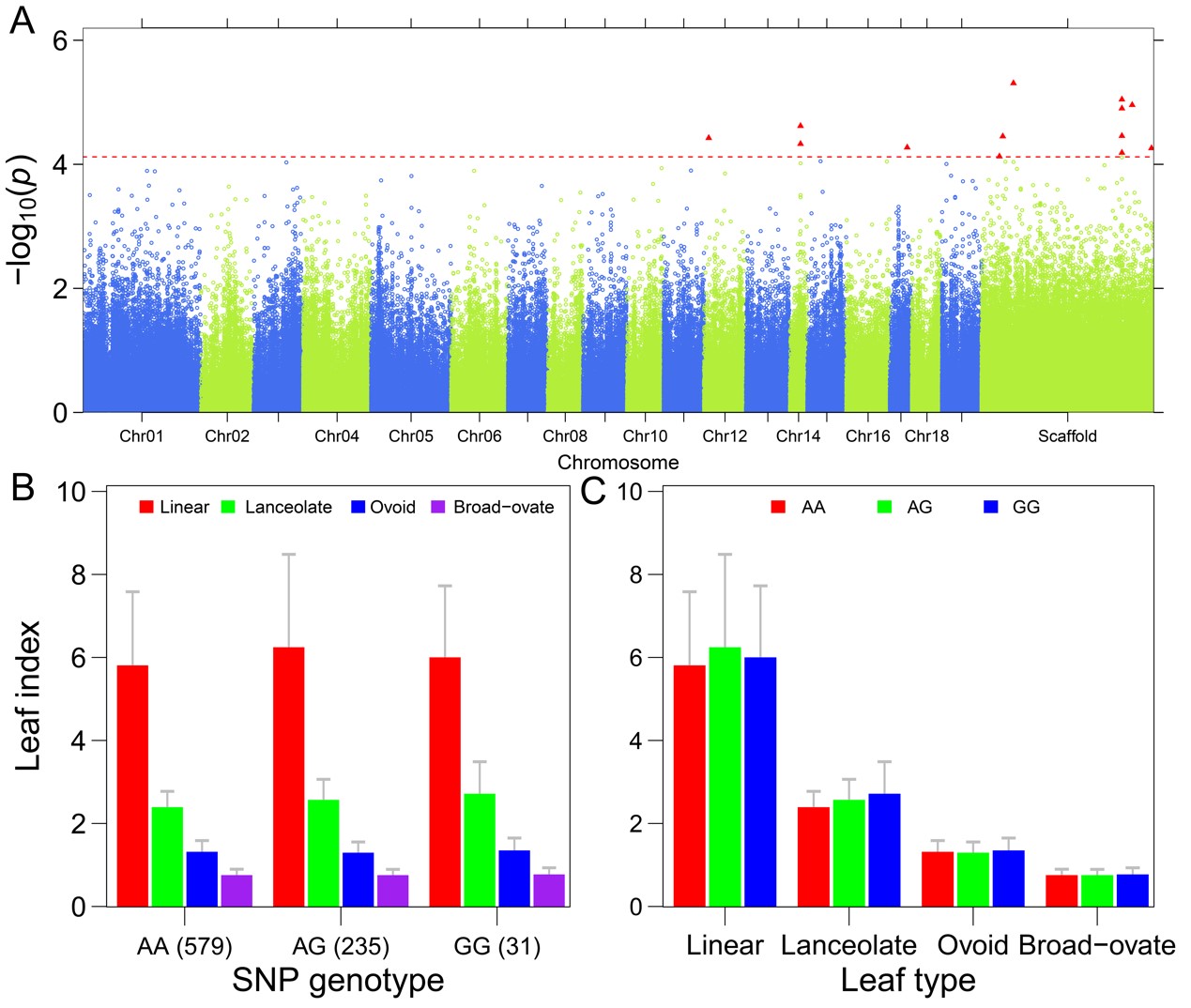


**Figure S9** Genome-wide association mapping of the leaf index in *P. euphratica*. (A) Manhattan plot displaying the GWAS result of the trait ‘leaf index’ based on the single-leaf GWAS. The significantly associated SNP markers are labeled. (B) Histograms of leaf length of different leaf types plotted as a function of genotypes at SNP262444. (C) Histograms of allelic effects of SNP262444 for the heterophylly.

**
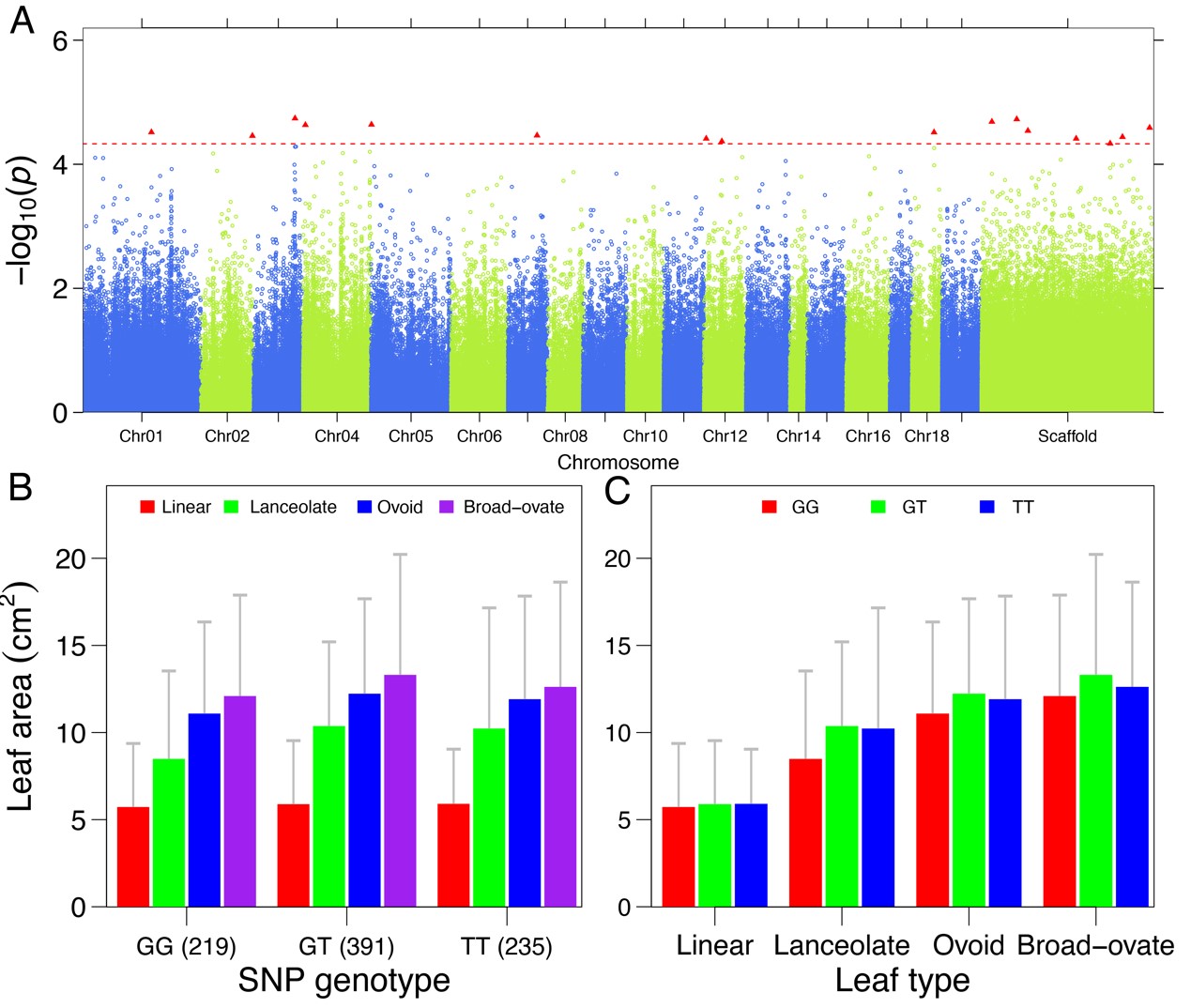
**

**Figure S10** Genome-wide association mapping of the leaf area in *P. euphratica*. (A) Manhattan plot displaying the GWAS result of the trait ‘leaf area’ based on the single-leaf GWAS. The significantly associated SNP markers are labeled. (B) Histograms of leaf length of different leaf types plotted as a function of genotypes at SNP57868. (C) Histograms of allelic effects of SNP57868 for the heterophylly.


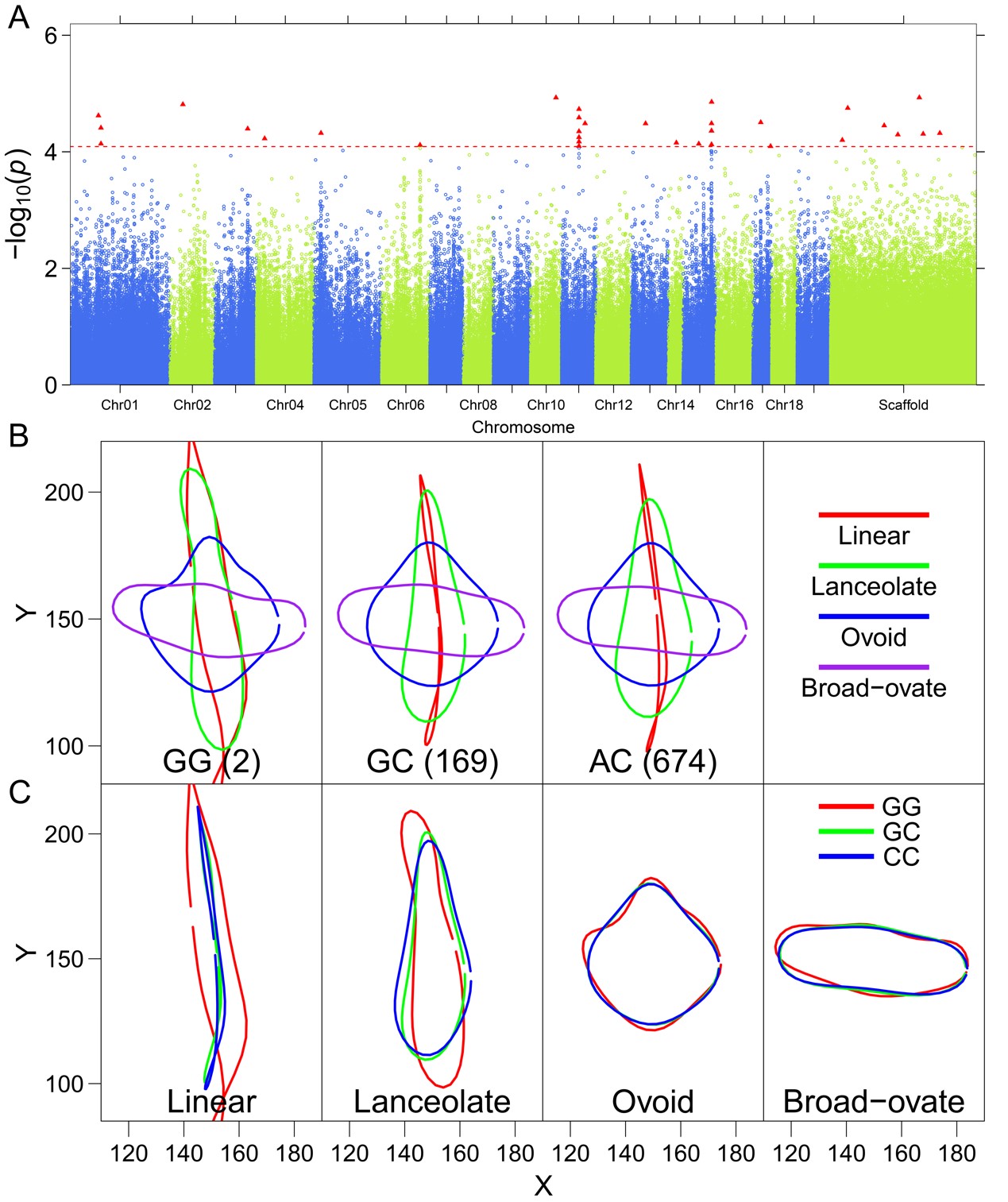


**Figure S11** Multi-leaf GWAS for the PC2 of leaf structure in *P. euphratica*. (A) Manhattan plot displaying the GWAS result of the PC2 based on the multi-leaf GWAS. The significantly associated SNP markers are labeled. (B) Leaf outline of PC2 of heterophylly plotted as a function of genotypes at SNP99561. (C) Visualized variation in the shape of four heterophyllous leaves in *P. euphratica* at a significant SNP (SNP99561 on scaffold NW_011499901.1).

**
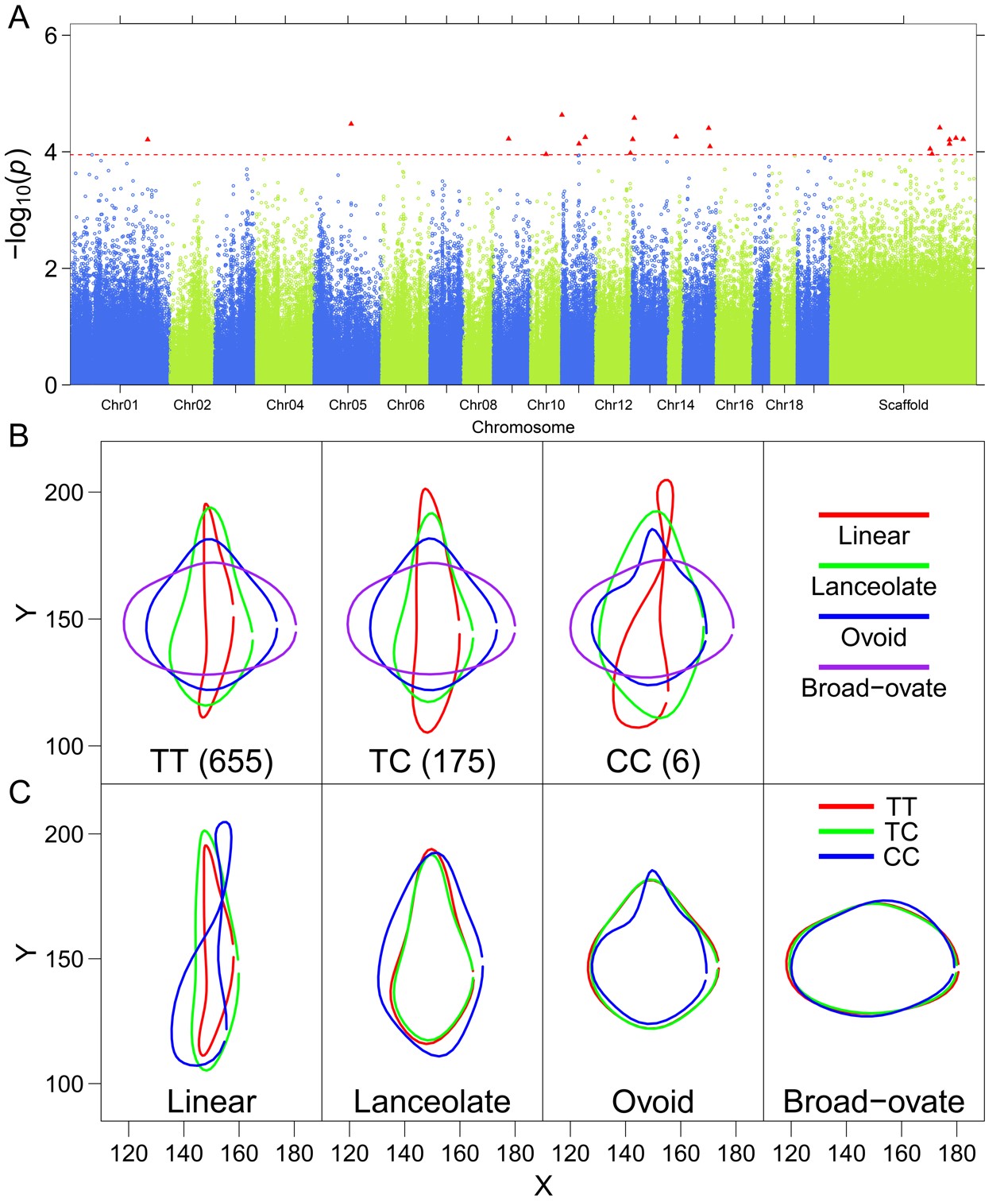
**

**Figure S12** Multi-leaf GWAS for the PC3 of leaf structure in *P. euphratica*. (A) Manhattan plot displaying the GWAS result of the PC3 based on the multi-leaf GWAS. The significantly associated SNP markers are labeled. (B) Leaf outline of PC3 of heterophylly plotted as a function of genotypes at SNP232072. (C) Visualized variation in the shape of four heterophyllous leaves in *P. euphratica* at a significant SNP (SNP232072 on scaffold NW_011500026.1).

**
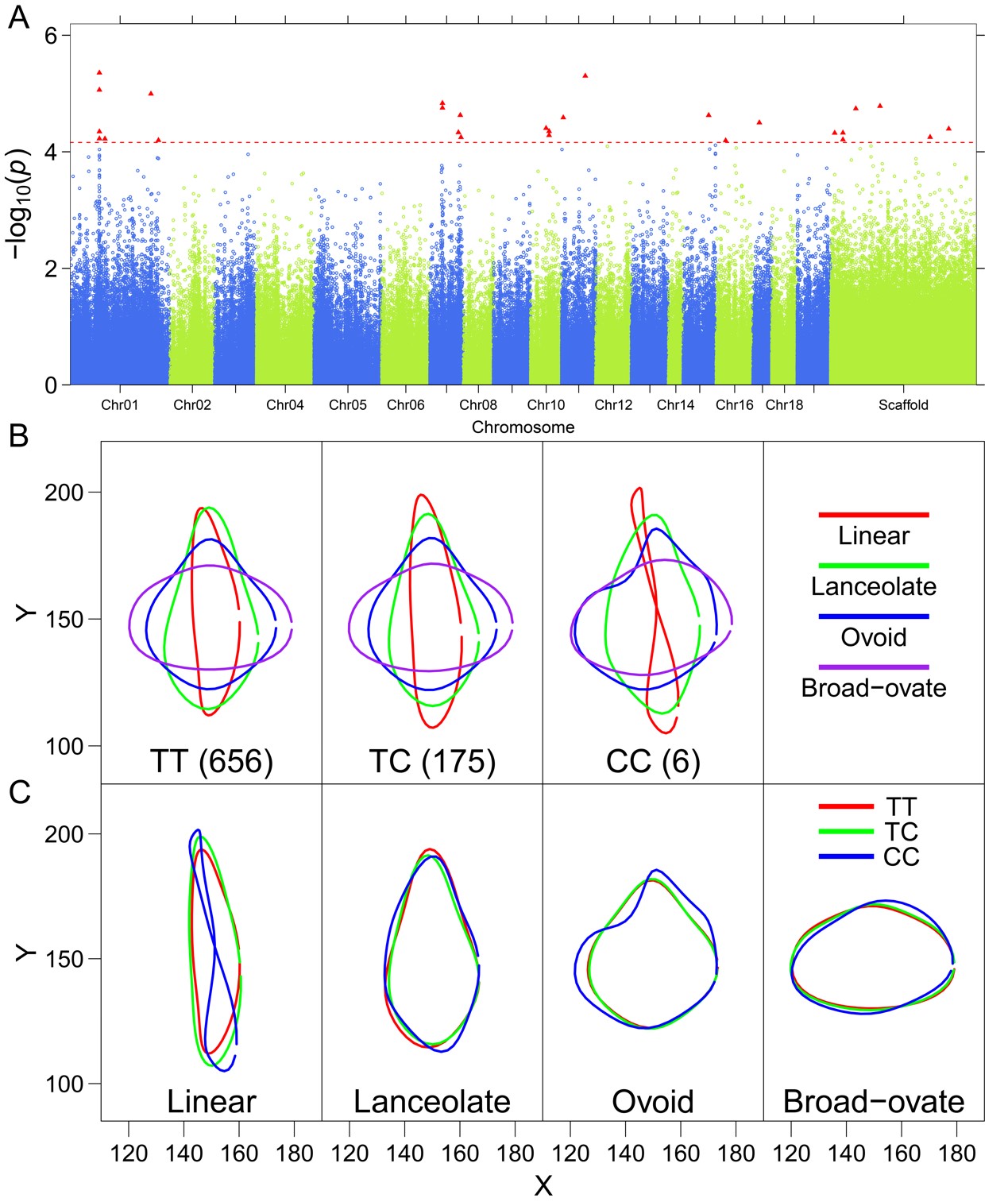
**

**Figure S13** Multi-leaf GWAS for the PC4 of leaf structure in *P. euphratica*. (A) Manhattan plot displaying the GWAS result of the PC4 based on the multi-leaf GWAS. The significantly associated SNP markers are labeled. (B) Leaf outline of PC4 of heterophylly plotted as a function of genotypes at SNP232072. (C) Visualized variation in the shape of four heterophyllous leaves in *P. euphratica* at a significant SNP (SNP232072 on scaffold NW_011500026.1).

**
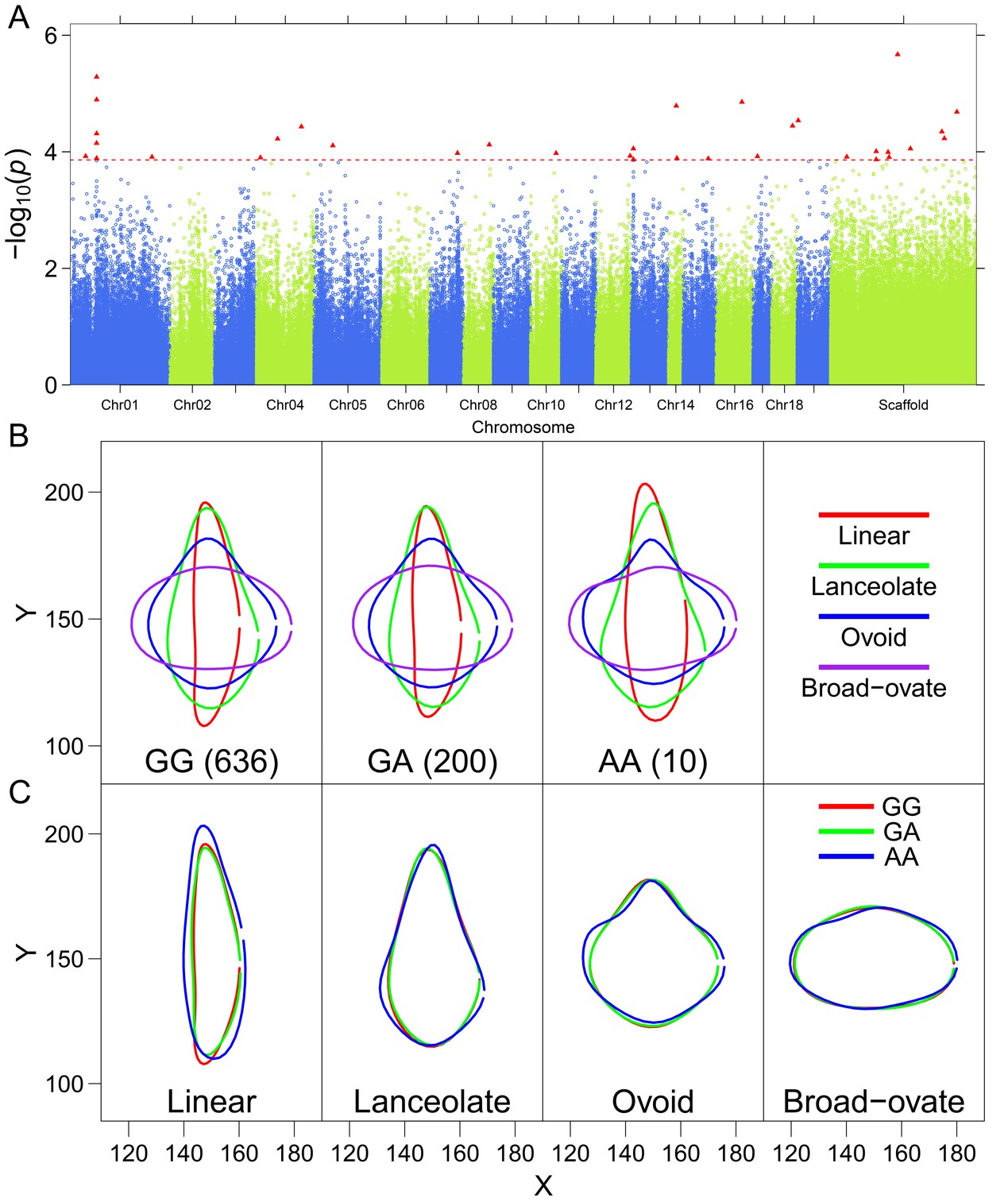
**

**Figure S14** Multi-leaf GWAS for the PC5 of leaf structure in *P. euphratica*. (A) Manhattan plot displaying the GWAS result of the PC5 based on the multi-leaf GWAS. The significantly associated SNP markers are labeled. (B) Leaf outline of PC5 of heterophylly plotted as a function of genotypes at SNP345841. (C) Visualized variation in the shape of four heterophyllous leaves in *P. euphratica* at a significant SNP (SNP345841 on scaffold NW_011500168.1).
